# Differential modulation of SARS-CoV-2 infection by complement factor H and properdin

**DOI:** 10.3389/fimmu.2025.1620229

**Published:** 2025-08-15

**Authors:** Uday Kishore, Praveen M. Varghese, Chandan Kumar, Susan Idicula-Thomas, Martin Mayora Neto, Anthony G. Tsolaki, Pretty Ponnachan, Khaled Masmoudi, Basel Al-Ramadi, Manu Vatish, Taruna Madan, Nigel Temperton, Nazar Beirag

**Affiliations:** ^1^ Department of Veterinary Medicine (CAVM), United Arab Emirates University, Al Ain, United Arab Emirates; ^2^ Zayed Centre for Health Sciences, United Arab Emirates University, Al Ain, United Arab Emirates; ^3^ Department of Clinical Microbiology, Umea University, Umea, Sweden; ^4^ Biomedical Informatics Centre, ICMR-National Institute for Research in Reproductive and Child Health, Mumbai, Maharashtra, India; ^5^ Viral Pseudotype Unit, Medway School of Pharmacy, University of Kent and Greenwich, Chatham, United Kingdom; ^6^ Department of Biosciences, College of Health, Medicine and Life Sciences, Brunel University London, Uxbridge, United Kingdom; ^7^ Department of Medical Microbiology and Immunology, College of Medicine and Health Sciences, United Arab Emirates University, Al Ain, United Arab Emirates; ^8^ Department of Integrative Agriculture (CAVM), United Arab Emirates University, Al Ain, United Arab Emirates; ^9^ ASPIRE Precision Medicine Research Institute Abu Dhabi, United Arab Emirates University, Al Ain, United Arab Emirates; ^10^ Nuffield Department of Women’s and Reproductive Health, University of Oxford, Oxford, United Kingdom; ^11^ Department of Innate Immunity, ICMR-National Institute for Research in Reproductive and Child Health, Mumbai, India

**Keywords:** innate immune system, complement system, alternative pathway, properdin, factor H, SARS-CoV-2, COVID-19, cytokine response

## Abstract

**Introduction:**

An unbalanced immune response and excessive inflammation are the major hallmarks of severe SARS-CoV-2 infection, which can result in multiorgan failure and death. The dysregulation of the complement system has been shown in various studies as a crucial factor in the immunopathology of SARS-CoV-2 infection. Complement alternative pathway has been linked to the excessive inflammation in severe SARS-CoV-2 infection in which decreased levels of factor H (FH) and elevated levels of properdin (FP) were observed. The current study investigated the potential immune protective roles of FP and FH against SARS-CoV-2 infection.

**Methods:**

The interactions between FH and FP and the SARS-CoV-2 spike (S) and its receptor binding domain (RBD) were evaluated using direct ELISA. The cell binding and luciferase-based viral entry assays utilising S protein expressing lentiviral pseudotypes were used to evaluate the possible modulatory effects of FH, FP, and recombinant thrombospondin repeats 4 and 5 (TSR4 + 5) on SARS-CoV-2 cell entry. Using RT-qPCR, we also assessed the immunomodulatory roles of FH and FP in the cytokine response induced by SARS-CoV-2 pseudotypes.

**Results:**

FH and FP were found to bind to both the RBD and SARS-CoV-2 S proteins. The treatment of FP or TSR4 + 5 enhanced cell binding and entry of SARS-CoV-2 pseudotypes that was administered in A549 cells expressing human ACE2 and TMPRSS2 (A549-hACE2+TMPRSS2 cells). FP increases the affinity between host ACE2 and SARS-CoV-2, according to *in silico* work. In A549-hACE2+TMPRSS2 cells, the effect of FP on viral cell entry and binding was counteracted by anti-FP antibody treatment. On the other hand, SARS-CoV-2 lentiviral pseudotypes’ cell entry and binding were decreased by FH treatment. The A549-hACE2+TMPRSS2 cells that were challenged with SARS-CoV-2 alphaviral pseudotypes (expressing spike, envelope, nucleocapsid, and membrane proteins) pre-treated with FP or TSR4+5 showed an upregulation of pro-inflammatory cytokine transcripts, including NF-κB and IL-1β, IL-8, IL-6, TNF-α, IFN-α, and RANTES. Contrary to this, the expression of these pro-inflammatory cytokines was downregulated by FH treatment. FH treatment decreased S protein-mediated NF-κB activation, but FP treatment enhanced it in A549-hACE2+TMPRSS2 cells.

**Discussion:**

These results imply that FH may function as a SARS-CoV-2 cell entry and binding inhibitor, reducing the inflammatory response linked to infection independently of complement activation. FP could aid cell viral entry and binding and aggravate hyperinflammation that might contribute to the severity of the infection.

## Introduction

The COVID-19 pandemic, caused by severe acute respiratory syndrome coronavirus type 2 (SARS-CoV-2), has had a significant global impact, with millions of infections and fatalities reported (1). The pathogenesis of COVID-19 is primarily characterised by dysregulation of the immune response (2). SARS-CoV-2 is an enveloped RNA virus of the Coronaviridae family (1). It consists of several structural proteins, including the nucleocapsid (N), membrane spike (S), membrane (M), and envelope (E) proteins, along with other auxiliary proteins that aid in viral entry and replication (1). The surface of SARS-CoV-2 is covered by the S protein, which comprises two subunits, S1 and S2, responsible for binding to host cell receptors (1). The virus infects various cell types, including alveolar macrophages and epithelial cells, by attaching to the angiotensin-converting enzyme 2 (ACE2) receptor (1).

The complement system plays a crucial role in both innate and acquired immunity, providing a robust defence against various infections caused by viruses, bacteria, fungi, and protozoa (3). There are three pathways of complement activation: classical, lectin, and alternative, all of which converge at the cleavage of complement component 3 (C3) (4). The alternative pathway distinguishes itself from the other two pathways by possessing a unique feature: a potent positive feedback loop that amplifies the activation of C3 regardless of the initial pathway involved (5). This amplification mechanism leads to an increased production of various pro-inflammatory effectors associated with complement (5). Notably, the alternative pathway is considered a driving force behind pathological complement activation, which contributes to the development of disease (5). A key factor in the regulation of the alternative pathway activation is properdin (FP), a positive regulator that facilitates and stabilises the assembly of C3bBb, the alternative pathway C3 convertase (6). Given that the alternative pathway is constitutively active at baseline and can also be activated because of classical/lectin pathway activation through the amplification loop, its regulation is crucial. In this regard, factor H (FH) plays a pivotal role in controlling the alternative pathway activity (7).

Previous studies on SARS-CoV have revealed its interaction with mannan-binding lectin (MBL), leading to the activation of the lectin pathway (8). Similarly, involvement of the complement system was shown in inducing hyperinflammation in middle east respiratory syndrome coronavirus (MERS-CoV) infection in animal models (9). Recently, attenuation of SARS-CoV-2 Infection has been shown by C1q and C4BP independently of complement activation (10).

SARS-CoV-2 has demonstrated the ability to activate the complement system through all three pathways (11). Dysregulation of the alternative pathway has been implicated in the severity and mortality associated with SARS-CoV-2 infection (12). Importantly, the alternative pathway can be directly activated by the SARS-CoV-2 S protein (13). Patients with severe COVID-19 exhibit abnormality in gene expression levels of key alternative pathway proteins, including FH and FP (12, 14–16). Complement FH is a soluble glycoprotein with a molecular weight of 155 kDa and functions as a negative regulator in the alternative pathway (17, 18). FH is present in human plasma at concentrations ranging from 128 to 654 µg/ml (17, 18). FH is synthesised by various immune and non-immune cells, including epithelial and endothelial cells (18, 19). A previous study has demonstrated interactions between FH and the soluble West Nile virus NS1 protein, suggesting a potential role for FH in the immune response against West Nile virus infection (20). Additionally, FH has been found to bind and inhibit influenza A virus (IAV) cell entry and attenuate inflammatory responses in A549 cells in a subtype-dependent manner (6). These findings highlight the importance of FH in the immune defence against viral infections.

Human FP is critical in stabilising C3 convertases by forming complexes such as C3bBbP and C3bBbC3bP (4). This stabilisation prolongs the half-life of these complexes and enhances the alternative pathway activity (4). Unlike most complement proteins that are primarily synthesised in the liver, FP is produced at a range of locations by immune active cells (4). Neutrophils are a major source of FP secretion but FP is also synthesised by monocytes and T cells (7). In human serum, FP exists as cyclic polymers, including cyclic dimers, trimers, and tetramers, with a plasma concentration ranging from 22 to 25 µg/ml (7). The monomeric form of FP consists of seven non-identical thrombospondin type 1 repeats (TSRs) and has a molecular weight of 53 kDa. Among these TSRs, TSR4 and TSR5 are believed to be crucial for binding to C3bBb (7). In recent studies, a recombinant form of TSR4 + 5, produced as a double domain, has been shown to bind C3b and inhibit the alternative pathway activity (7). Moreover, FP has been found to inhibit IAV (H1N1 subtype) cell entry and binding as well as attenuate the pro-inflammatory immune response in a complement activation - independent manner (7).

To date, the immune functions of FH and FP have not been explored in the context of SARS-CoV-2 infection, independent of the complement activation. This study investigates the roles of human FH, FP, and recombinant TSR4 + 5 against SARS-CoV-2 infection, specifically focusing on mechanisms that operate independently of complement activation. The interaction between FH and FP and the RBD and of the SARS-CoV-2 spike (S) protein was examined. Using a model system with A549 cells expressing human ACE2 and TMPRSS2 receptors to mimic SARS-CoV-2 infection, the potential of FH and FP to limit viral entry was evaluated.

Our findings demonstrate that FH can effectively inhibit SARS-CoV-2 pseudotypes cell entry, independent of complement activation, while FP enhances it. Moreover, we observed that treatment with FH resulted in the downregulation of the proinflammatory response triggered by SARS-CoV-2, whereas FP upregulated it

## Materials and methods

### Purification of human complement factor H

Human complement FH was isolated from human plasma using an affinity column on a monoclonal antibody against human FH (MRCOX23) coupled to CNBr-activated Sepharose (GE Healthcare, UK) as described previously (21). Freshly thawed mixed human plasma (50ml) (TCS Biosciences) was adjusted to a concentration of 5mM EDTA, pH 8 and dialysed against buffer I (25 mM Tris-HCL, 140 mM NaCl, and 0.5 mM EDTA at pH 7.5) stirring overnight at 4°C. Dialysed plasma was passed through the MRCOX23 Sepharose column after it had been washed with 5-bed volumes of buffer I. The filtered plasma was passed through the column and washed with the same buffer. FH was then eluted using 3M magnesium chloride (Merck), 25 mM Tris pH 8.0, 140 mM NaCl, and 0.5 mM EDTA. The collected fractions (1 ml) were neutralised using 1 M Tris pH 7.5. The fractions were then dialysed against 1 L of distilled water overnight. The next day, the fractions were dialysed against H_2_O overnight, followed by 10 mM potassium phosphate for 4h. Western blotting was performed to confirm the isolated protein (**Supplementary Figure S1A**). A yield of ~ 1.5mg/ml was obtained; an approximate recovery of 6%, based on an estimated total of 25 mg of FH in 50 mL of human plasma (22).

### Purification of native human properdin

Human native FP was purified using the affinity column, as previously mentioned (23, 24). Briefly, 100ml of freshy thawed non-sterile mixed pool human plasma was adjusted to a final concentration of 5 mM EDTA, pH 8. In order to exclude contamination risks the mixed pool human plasma was centrifuged at 5000 x g and filtered using a Whatman filter paper.

An IgG-Sepharose column was washed with 3-bed volumes of HEPES buffer (10 mM HEPES, 140 mM NaCl, 0.5 mM EDTA, pH 7.4). The plasma was then passed through the IgG-Sepharose column to remove C1q. The plasma depleted of C1q was then passed through the anti-properdin column and washed with 3-bed volumes of HEPES buffer. Further, the bound FP was eluted using 3M MgCl2. The eluted fractions (1 ml) were dialysed against HEPES buffer, stirring overnight at 4°C. Finally, to ensure quality of sample, the fractions were passed through the HiTrap Q FF-Sepharose ion exchange column (GE Healthcare, UK) to remove any impurities. Western blotting was used to evaluate immunoreactivity of the samples (**Supplementary Figure S1B**). A yield of ~ 1.2mg/ml was obtained from 100 ml plasma; approximating a 48% recovery based on an expected total of 2.5 mg in human plasma (25).

### Expression and purification of properdin-TSR4 + 5

As previously described (23, 24). recombinant TSR4 + 5 nodule was expressed and produce in E. coli BL21 cells as a recombinant protein and fused to maltose-binding protein (MBP) using an amylose resin column. 500 mL LB media culture, containing 100 μg/mL of ampicillin, was inoculated with 12.5 mL of a protein-expressing starter culture and was allowed to mix briefly. The culture was incubated at 37°C for 3h in a shaker until the OD600 reached 0.6. The bacterial cell pellet was resuspended in 25 mL of lysis buffer comprising 20 mM Tris-HCl (pH 8.0), 0.5 M NaCl, 1 mM EGTA (pH 7.5), 1 mM EDTA (pH 7.5), 5% v/v glycerol, 0.2% v/v Tween 20, 0.1 mM PMSF, and 50 μg/mL lysozyme followed by centrifugation at 13,800 x g for 10 min. The resuspended cells were then incubated at 4°C for 30 min. The lysate was subjected to 12 cycles of sonication at 60 Hz for 30 seconds each, separated by a 2-minute gap. Subsequently, the lysate was centrifuged for 30 minutes at 4°C at 15,000 x g. The supernatant was diluted with 125 mL of buffer I, which contains 0.2% v/v Tween 20, 5% v/v glycerol, 1 mM EDTA (pH 7.5), 100 mM NaCl, and 20 mM Tris-HCl (pH 8.0) and was purified by passing it through a 5 mL amylose resin column (New England Biolabs). 150 mL of buffer I and 250 mL of buffer II (buffer I without Tween 20) were used to wash the column. 100 mL of buffer II containing 100 mM maltose was used to elute the fusion protein. Finally, PierceTM High-Capacity Endotoxin Removal Resin (Qiagen, Hilden, Germany) was used to remove lipopolysaccharides (LPS) contaminants. The QCL-1000 Limulus amebocyte lysate technology (Lonza, Basel, Switzerland) to was used to measure the endotoxin levels in the purified protein samples, found to be about 4 pg/μg. Western blotting was used to identify the isolated proteins (**Supplementary Figure S1C**). The purification yield about 2 mg of purified protein.

### Cell culture

Adenocarcinoma human alveolar basal epithelial cells (A549) expressing co-receptors; human ACE2 and TMPRSS2 were cultured as previously as reported (10). A549 cells were cultured in Dulbecco’s Modified Eagle’s Medium (DMEM) containing Glutamax (Gibco), which was supplemented with 10% v/v foetal bovine serum (FBS) (Gibco) and 100U/ml penicillin (Gibco) as complete growth media. The culture was maintained at 37°C with 5% v/v CO_2_. Using FuGENETM HD Transfection Reagent (Promega), the cells were transiently co-transfected with two plasmids: (pCAGGS-TMPRSS2) expressing TMPRSS2 and (pCDNA3.1+-ACE2) expressing human ACE2. The following day, selection for cells co-expressing both human ACE2 and TMPRSS2 (A549-hACE2+TMPRSS2 cells) was carried out by incubation of cells in media supplemented with hygromycin and puromycin (Thermo Fisher Scientific, Waltham, MA, USA).

### ELISA

To determine the efficiency of attachment by SARS-CoV-2 S or RBD proteins to immobilised FH or FP, decreasing concentrations of immobilised FH or FP (1, 0.5, 1.25, and 0 μg/well) were coated on polystyrene microtiter plates (Sigma-Aldrich) using carbonate/bicarbonate (CBC) solution, pH 9.6, followed by overnight incubation at 4°C. MBP (1μg/well) was used as a negative control. The following day, the wells were rinsed thrice using PBST Buffer (PBS + 0.05% Tween 20) (Fisher Scientific) to get rid of unbound proteins. Further, the wells were blocked for 2h at 37°C using 2% w/v BSA in PBS (Fisher Scientific) followed by three PBS washes. The corresponding wells coated with FH or FP were treated with a constant dose of 1μg/well in 100 μl of recombinant SARS-CoV-2 S protein (RP-87680, Invitrogen) or recombinant SARS-CoV-2 spike RBD protein (40592-V08H, Sino-Biological).

To investigate the binding of the FH or FP to the immobilised SARS-CoV-2 S or RDB, a second experiment was conducted. A decreasing dose of FH or FP (1, 0.5, 1.25, and 0 μg/well) were added to immobilised SARS-CoV-2 S (1 μg/100 μl) or RBD (1 μg/100 μl) coated wells. After blocking the wells with 2% w/v BSA in PBS for 2h at 37°C, the wells were again washed 3 times using PBST.

Binding interactions between the viral S protein and the immobilised FH, FP or MBP were detected using polyclonal rabbit anti-SARS-CoV-2 S antibodies. For detection of the bound host proteins to the immobilised S protein; monoclonal mouse anti-human FH antibody (MRCOX23) was used for FH, and rabbit anti-human properdin polyclonal antibody for FP. All primary antibodies were used at a 1:5000 dilution and incubated for 1h at 37°C.

Subsequently, appropriate HRP-conjugated secondary antibodies (goat anti-rabbit IgG or goat anti-mouse IgG) were added at 1:5000 dilution and incubated for 1h at 37°C. Following final washes, TMB substrate was applied, and the enzymatic reaction was stopped with 1M sulfuric acid. Absorbance was measured at 450 nm using an iMark™ microplate absorbance reader.

### Viral cell entry assay

#### Treatment of SARS-CoV-2 spike protein pseudotyped lentiviral particles

Pseudotyped lentiviral particles containing the SARS-CoV-2 S lentiviral particles with were generated as previously reported (26). A549-hACE2+TMPRSS2 cells were challenged with SARS-CoV-2 lentiviral pseudoparticles that had been pre-incubated with 20 μg/ml FH, FP (with or without 20 μg/ml anti-human FP polyclonal antibodies), or TSR4 + 5 for 2h at room temperature (RT). Cells challenged with only lentiviral pseudoparticles served as controls.

#### Luciferase assay

An assay for infection utilising Luciferase reporter activity was performed to investigate potential effects of FH, FP, or TSR4 + 5 treatment on SARS-CoV-2 cell entry. In brief, complete growth media was used to seed A549-hACE2+TMPRSS2 cells (20,000 cells/well) on a 96-well plate and let them to adhere overnight at 37°C. The following day, the cells were challenged with FH, FP, or TSR4 + 5-treated SARS CoV-2 lentiviral pseudoparticles. These cells were then incubated for 24h at 37°C in serum-free growth media (DMEM with Glutamax supplemented with 100U/ml penicillin and 100μg/ml streptomycin) after which they were then washed twice with PBS followed by addition fresh growth media, and were further incubated 48h at 37°C. Finally, the luciferase activity (RLU) was evaluated using the ONE-GloTM Luciferase Assay System (Promega) and Clariostar Plus Microplate Reader (BMG Labtech), after cells were washed.

#### NF-κB activity assay

Determination of the modulation potential of FH and FP on NF-κB activity during SARS-CoV-2 infection was estimated using a luciferase-based reporter assay. In short, A549-hACE2+TMPRSS2 cells was transfected with pNF-κB-LUC plasmid (#631904, Clonetech). The latter contains several copies of the NF-κB consensus sequences fused to a TATA-like promoter region of the Herpes Simplex Virus Thymidine Kinase (HSV-TK) promoter. The plasmid is designed to quantify the transcription factor’s binding to the κ enhancer, enabling a direct assessment of this pathway. After endogenous NF-κB binds to the κ enhancer element, transcription is induced, and the reporter gene is activated. The A549 cells were transfected with the plasmid using the Promega FuGENETM HD Transfection Reagent, then cultured for 48h at 37°C in complete growth media. Post transfection, 20,000 cells per well were seeded in a 96-well plate and allowed to adhere overnight at 37°C. The next day, the cells were challenged with 500ng/ml of SARS-CoV-2 S protein pretreated with 20 μg/ml FH or FP (for 2h at RT) and incubated for 24h in serum-free growth media at 37°C. As a control, A549-hACE2+TMPRSS2 cells+SARS-CoV-2 S protein were used. The luciferase reporter assay for NF-κB activity was performed as mentioned above.

### Cell binding assay

The impact of FH or FP treatment on SARS-CoV-2 cell binding competency, was evaluated using a cell binding assay. Briefly, 20,000 of A549-hACE2+TMPRSS2 cells were plated in a 96-well plate and allowed to adhere overnight at 37°C in growth media. The next day, the cell was challenged with SARS-CoV-2 lentiviral pseudoparticles pre-treatment with FH, FP or TSR4 + 5 (20 μg/ml) and incubated for 2h at 37°C in serum-free growth media. This assay also involved FP sequestration, pseudoparticles were co-incubated with anti-human properdin polyclonal antibody to block FP-mediated effects. An anti-SP-D antibody was used as a negative antibody control. After incubation, the cells were washed thrice with PBS and was fixed using 1% v/v paraformaldehyde (PFA) at RT for 1 min. A final series of three consecutive PBS washes were performed before probing the wells with Alexa Fluor 488 conjugated goat anti-rabbit antibody (1:200) (Abcam) for 1h at RT. Clariostar Plus Microplate Reader (BMG Labtech) was used to read the plate.

### Estimating FH/FP modulated cytokine response during SARS-CoV-2 infection

#### SARS-CoV-2 alphaviral pseudoparticles treatment

SARS-CoV-2 alphaviral pseudoparticles that encoded the four structural proteins, S, E, M, and N (Ha-CoV-2 Luc; Virongy, Manassas, VA, USA), were utilised for gene expression analysis (RT-qPCR). Alphaviral pseudoparticles were pre-incubated for two hours at RT with 20μg/ml FH, FP, or TSR4 + 5. 20μg/ml MBP used as negative control. These pre-treated alphaviral pseudoparticles were used to challenge A549-hACE2+TMPRSS2. The untreated control group consisted of cells that were challenged with pseudoparticles but had not previously been incubated with complement proteins.

### Quantitative qRT-PCR analysis

The potential effect of FH or FP treatment on pro-inflammatory gene expression levels in cells exposed to SARS-CoV-2 pseudoparticles was evaluated using a qRT-PCR. Briefly, A549-hACE2+TMPRSS2 cells (0.5 X 10^6^) were seeded in a 12-well dish supplemented with adequate growth media and incubated overnight at 37°C and 5% v/v CO_2_ and confirmed for cell attachment the following day. SARS-CoV-2 alphaviral pseudoparticles pre-treated with 20μg/ml FH, FP, or TSR4 + 5 were added to A549-hACE2+TMPRSS2 cells the following day, and they were cultured for 6 and 12 hours at 37°C in serum-free growth media. After that, the cells were cleaned with PBS and pelleted. The total RNA was extracted using the GenElute Mammalian Total RNA Purification Kit (Sigma-Aldrich), in accordance with the manufacturer’s instructions. To guarantee the removal of any potential genomic DNA contamination, the extracted RNA samples were treated with DNase I (Sigma-Aldrich). RNA concentrations were measured at 260 nm wavelength using a ThermoFisher Nanodrop 2000/2000c. The A260/A280 ratio was used to determine the RNA’s purity. 2 µg of total RNA extracted was used to synthesise cDNA using the High-Capacity RNA to cDNA Kit (Applied Biosystems).

The primer BLAST programme (Basic Local Alignment Search Tool) was used to create the primer sequences (**Table 1**). Applied Biosciences’ Step One Plus equipment was used to conduct the qRT-PCR experiment. 500 ng of cDNA, 75 nM forward and reverse primers, and 5 μl of Applied Biosystems’ Power SYBR Green Master Mix were used in each triplicate qPCR experiment. After running the qPCR samples for two and ten minutes at 50°C and 95°C, the amplification template was run for forty cycles, with 15 seconds at 95°C and one minute at 60°C in each cycle. To normalise the expression of the genes, 18S rRNA was used as an endogenous control.

**Table 1:** Forward and reverse primers used for qRT-PCR assay.

Gene	Forward primer	Reverse primer
18S	5′-ATGGCCGTTCTTAGTTGGTG-3′	5′-CGCTGAGCCAGTCAGTGTAG-3′
TNF-α	5′-AGCCCATGTTGTAGCAAACC-3′	5′-TGAGGTACAGGCCCTCTGAT-3′
IL-6	5′-GAAAGCAGCAAGAGGCACT-3	5′-TTTCACCAGGCAAGTCTCCT-3′
IL-8	5′-GTGCAGTTTTTGCCAAGGAG-3′	5′-CACCCAGTTTTCCTTGGGGT-3′
NF-κB	5′-GTATTTCAACCACAGATGGCACT-3′	5′-AACCTTTGCTGGTCCCACAT-3′
RANTES	5′-GCGGGTACCATGAAGATCTCTG-3′	5′-GGGTCAGAATCAAGAAACCCTC-3′
IFN-α	5′-TTTCTCCTGCCTGAAGGACAG-3′	5′-GCTCATGATTTCTGCTCTGACA-3′
IL-1β	5′-GTGCAGTTTTGCCAAGGAG-3′	5′-ACGTTTCGAAGATGACAGGCT-3′

### *In silico* interaction analysis involving FP, spike and ACE2 receptor

The binding site prediction in S protein for FP was performed using a blind docking approach. Structural coordinates for FP monomer (domains 1, 4, 5 & 6) and S trimer were retrieved from RCSB with PDB IDs 6S08 and 6XM3, respectively. The electron microscopy structure of S bound to ACE2 receptor (PDB ID: 7KNB) was retrieved and the inter-molecular interactions were analysed. The interacting residues were used to define the binding site for re-docking experiment with the aim to generate the binding energy of the docked complex. The previously docked complex of spike and FP, along with the ACE2 receptor structure, extracted from PDB ID: 7KNB, was used to generate a tripartite complex structure of spike, FP and ACE2. All proteins were prepared for docking using the ‘Prepare Proteins’ module of Discovery Studio (DS) 2021 with default parameters setting. ‘ZDOCK’ module of DS 2021 was used for docking with default parameters and docked poses were further refined using ‘ZRank’ algorithm. Top cluster poses were analysed, and the final pose was selected based on concurrence with known interaction of ACE2 with spike (as present in PDB ID: 7KNB), and maximal interaction of domains 4 (TSR4) and 5 (TSR5) of FP with spike. Binding free energy for three complexes, (a) spike and FP, (b) spike and ACE2, and (c) spike-FP and ACE2 were calculated using in-house ‘Binding Free Energies’ protocol in DS 2021.

An attempt was made to predict the intermolecular interactions of FH, spike, and ACE2 receptor. This experiment could not be performed due to unavailability of full-length 3D structure of FH in PDB. The Alpha-fold (ID: AF-P08603) or homology based modelled structure that was generated for FH was sub-optimal due to several unfolded regions and thus, was not fit for the docking studies (**Supplementary Figure S3**).

### Statistical analysis

The graphs were made with the aid of GraphPad Prism 9.0. According to the figure legends, the statistical significance between the treated and untreated situations was considered. The SD or SEM that the error bars reflect is stated in the figure legends.

## Results

### SARS-CoV-2 spike and RBD proteins bind to human FH and FP

**Figure 1:**
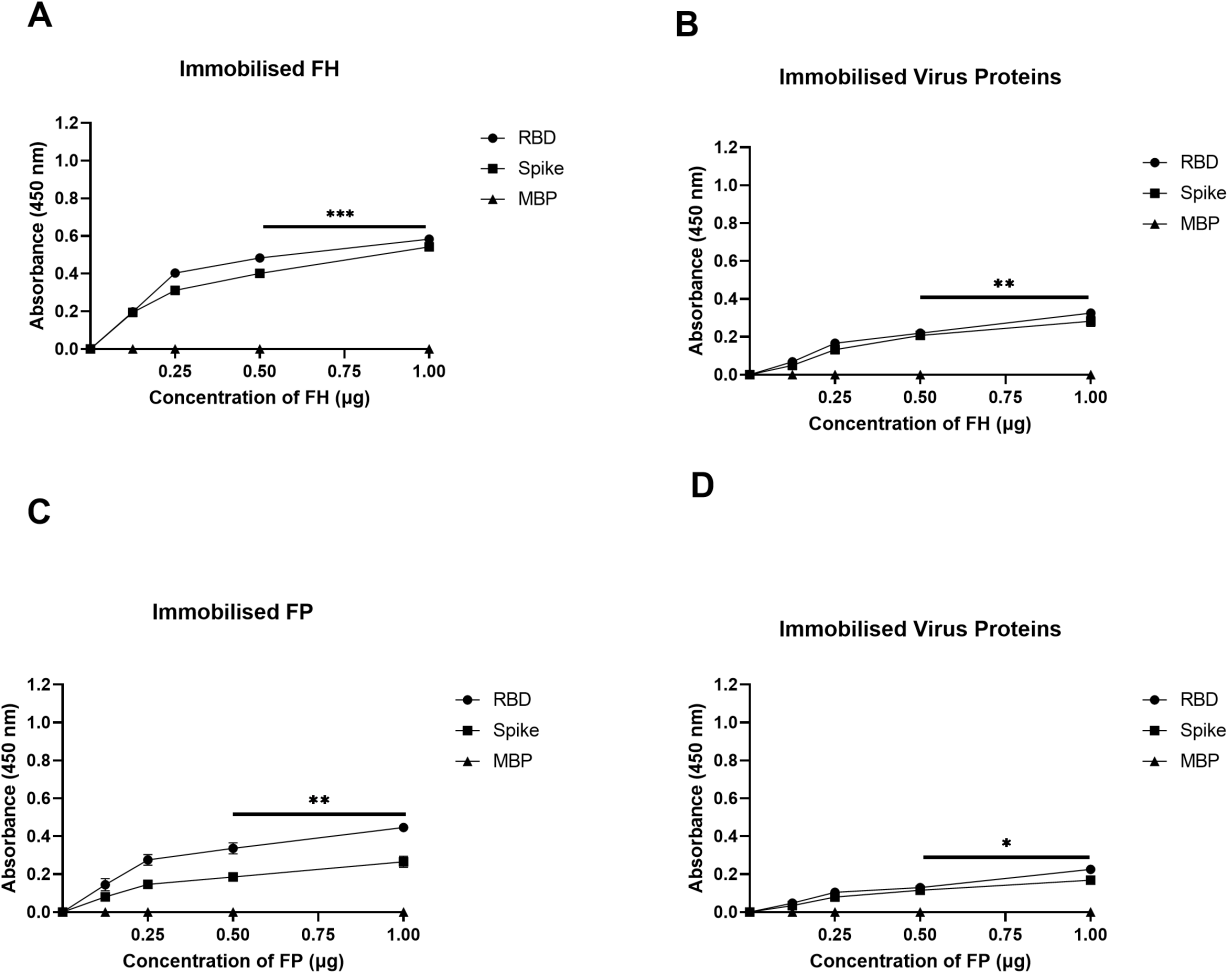
SARS-CoV-2 S protein directly interacts with FH and FP via its RBD. FH bound both SARS-CoV-2 S and RBD proteins in a dose-dependent manner. Decreasing concentration of FH or FP (1, 0.5, 1.25, and 0 μg/well) **(A, C)**, or constant concentration of viral proteins (1 μg/well) **(B, D)** were immobilised using a 96-well plate using Carbonate-Bicarbonate (CBC) buffer, pH 9.6 at 4°C overnight. After washing off the excess CBC buffer with PBS, a constant concentration of viral proteins (1 μg/well) **(A, C)**, or decreasing concentrations of FH or FP (1, 0.5, 1.25, and 0 μg/well) **(B, D)** were added to the corresponding wells, followed by incubation at 37°C for 2h. After washing off the unbound proteins, the wells were probed with corresponding primary antibodies (1:5000; 100 μl/well), i.e. rabbit anti-SARS-CoV-2 S or rabbit anti-human FH or FP polyclonal antibodies. BSA was used as a negative control. The data are presented as a mean of three independent experiments carried out in triplicates ± SEM. Significance was determined using the one-way ANOVA test (*p 0.05, **p<0.01 and ***p < 0.001).

A direct ELISA assay was employed to investigate the binding interactions between purified FH and SARS-CoV-2 S and RBD proteins (**Figure 1A**), as well as the reciprocal binding of SARS-CoV-2 S and RBD proteins to immobilised FH (**Figure 1B**). The results revealed a dose-dependent binding of FH to S and RBD proteins when probed with the anti-SARS-CoV-2 S protein polyclonal antibody. Similarly, immobilised S protein or RBD displayed a dose-dependent binding to FH when probed with the rabbit anti-human FH polyclonal antibody.

The binding capacity of purified FP to SARS-CoV-2 S and RBD proteins was assessed similarly (**Figure 1C**), along with the reciprocal binding of SARS-CoV-2 S and RBD proteins to immobilised FP (**Figure 1D**). The results demonstrated that immobilised FP exhibited dose-dependent binding to S and RBD proteins when probed with the polyclonal anti-SARS-CoV-2 S protein antibody. Similarly, the immobilised SARS-CoV-2 S or RBD proteins showed dose-dependent binding to FP when probed with the rabbit anti-human properdin polyclonal antibody. MBP was used as a negative control protein in the assay. A comparable result was obtained using recombinant TSR4 + 5 modules when probed with anti-MBP antibodies (**Supplementary Figure S2**).

### FH restricted SARS-CoV-2 Pseudoparticle transduction while FP and TSR4 + 5 promoted

**Figure 2:**
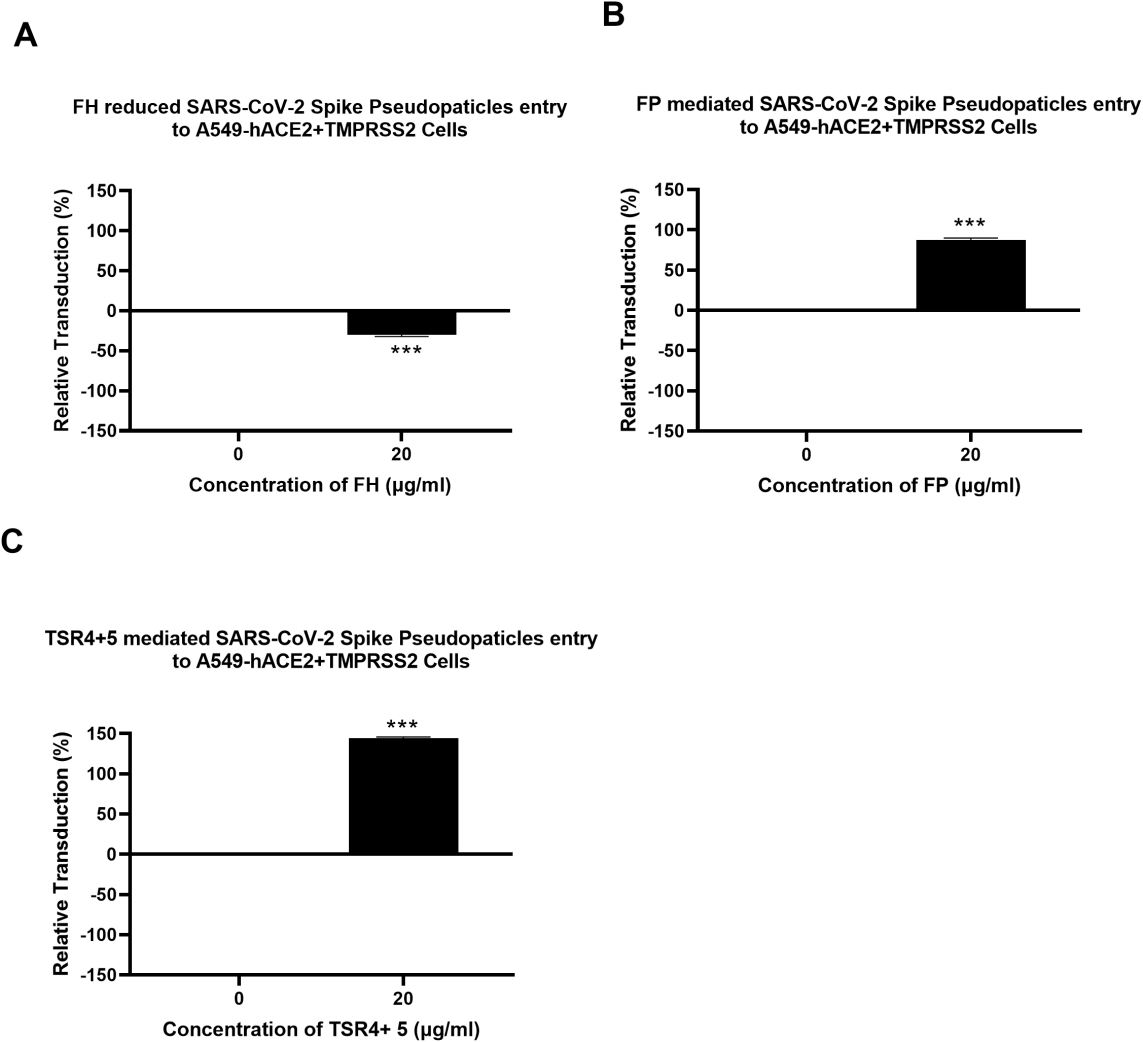
Modulation of SARS-CoV-2 pseudotype viral entry in A549-hACE2+TMPRSS2 cells by FH, FP or TSR4 + 5 treatment. FH, FP or TSR4 + 5 (20μg/ml) were used to pre-treat SARS CoV-2-lentiviral pseudoparticles **(A–C)**, respectively. To determine if the treatment affected the virus capacity to enter the cells, either treated or untreated lentiviral pseudoparticles were transduced into A549-hACE2+TMPRSS2 cells and then examined for luciferase reporter activity. The background was subtracted from all data points. The data obtained were normalised with 0% luciferase activity defined as the mean of the relative luminescence units recorded from the control sample (Cells + lentiviral pseudoparticles). Data are shown as the normalised mean of three independent experiments done in triplicates ± SEM. Significance was determined using the two-way ANOVA test (***p < 0.001) (n = 3).

A luciferase reporter assay was conducted to assess the impact of FH, FP and TSR4 + 5 on SARS-CoV-2 infectivity. SARS-CoV-2 lentiviral pseudoparticles, pre-treated with FH showed reduced viral transduction in A549-hACE2+TMPRSS2 cells while increased transduction was observed in the case of FP or TSR4 + 5 pretreated pseudoparticles compared to their respective controls. Specifically, A549-hACE2+TMPRSS2 cells challenged with SARS-CoV-2 lentiviral pseudoparticles pre-treated with FH, significantly reduced viral infection by ~25% (**Figure 2A**). FP and TSR4 + 5 treatment increased viral entry by ~80% and ~140% (**Figures 2B, C**), respectively, compared to their respective controls. These findings suggested that FH acted as an entry inhibitor for SARS-CoV-2 pseudotyped particles, whereas FP facilitated viral entry.

### SARS-CoV-2 pseudoparticle binding to the target cells was inhibited by FH and enhanced by FP and TSR4 + 5

**Figure 3:**
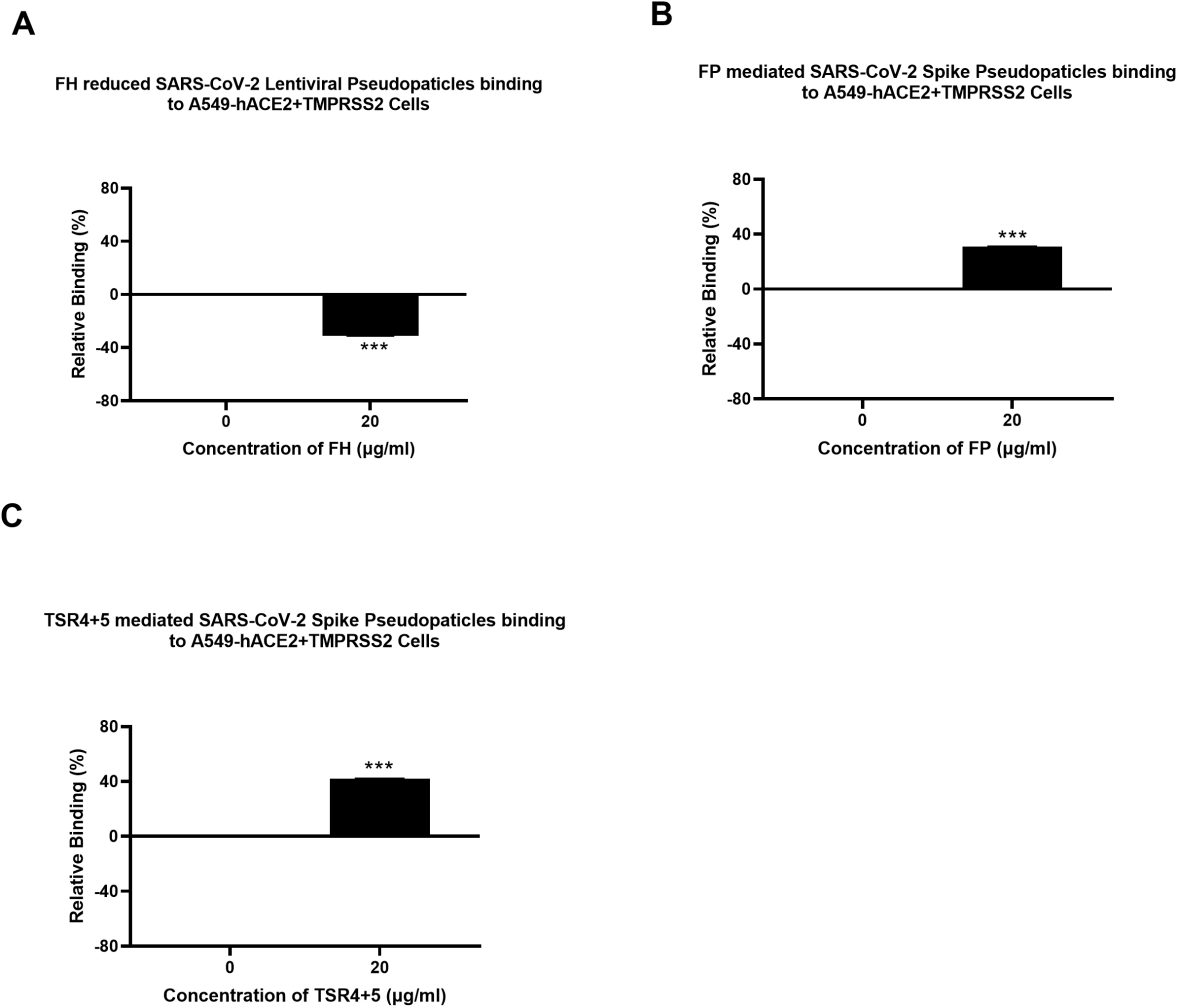
Modulation of SARS-CoV-2 pseudoparticle binding to A549-hACE2+TMPRSS2 cells by FH, FP or TSR4 + 5. Cell binding assay revealed FH treatment inhibits the binding to the cell-surface receptor, whereas FP and TSR4 + 5 mediated it **(A–C)**, respectively. A549-hACE2+TMPRSS2 cells (1 × 10_5_ cells/ml) were challenged with SARS-CoV-2 lentiviral pseudoparticles pre-incubation with or without FH, FP or TSR4 + 5 (20 μg/ml), followed by incubating at 37°C for 2h. After removing unbound protein and viral particles, the wells were fixed with 1% v/v PFA for 1 min and probed with polyclonal rabbit anti-SARS CoV-2 S (1:200). The data obtained were normalised with 0% fluorescence defined as the mean of the relative fluorescence units recorded from the control sample (Cells + lentiviral pseudoparticles). Three independent experiments were carried out in triplicates; error bars are expressed as ± SEM. Significance was determined using the two-way ANOVA test (***p < 0.001) (n = 3).

**Figure 4:**
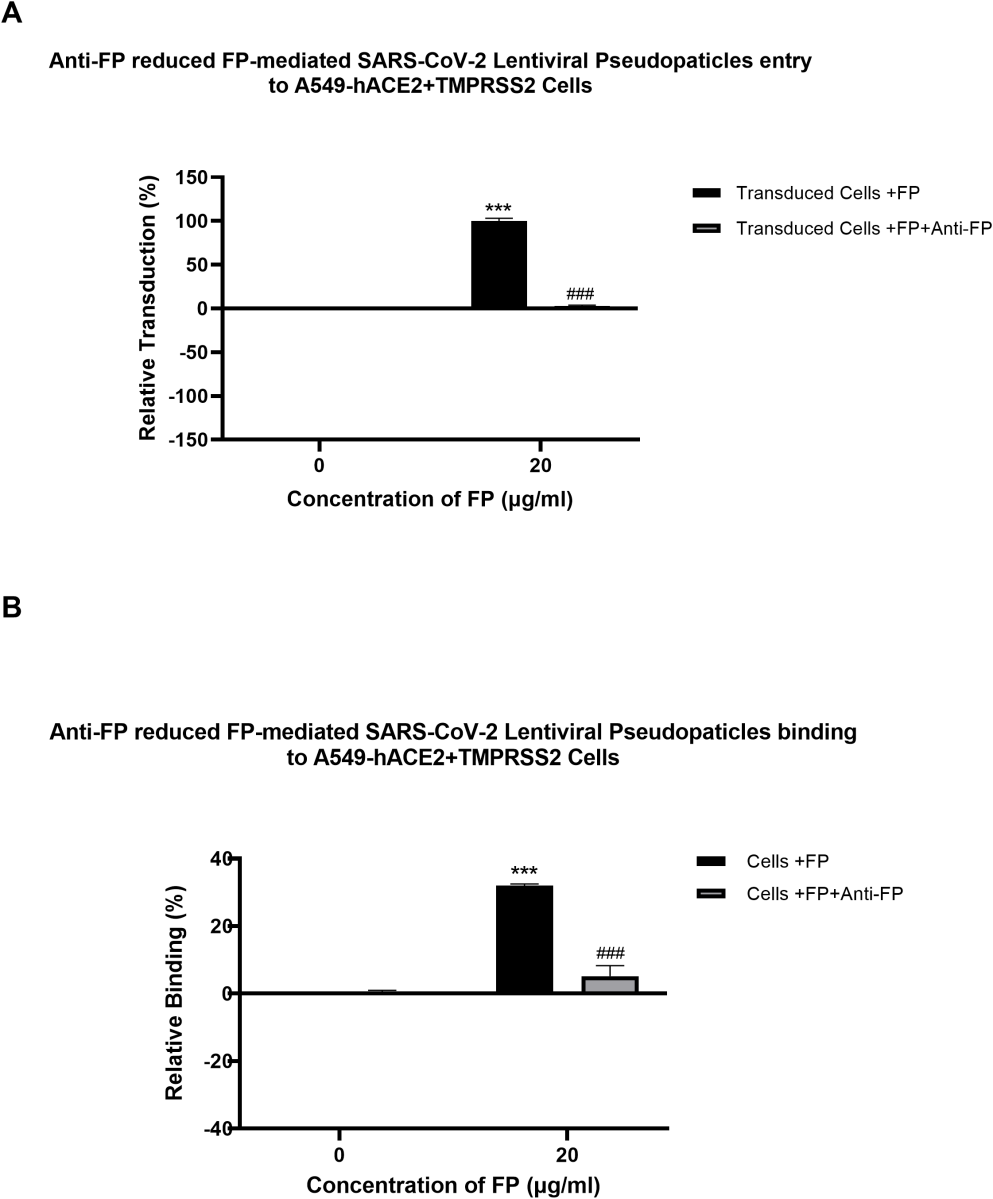
Reversal of FP mediated-SARS-CoV-2 viral entry **(A)** and binding **(B)** to A549-hACE2+TMPRSS2 cells by anti-FP antibody. FP-treated (with or without anti-FP antibodies) or untreated lentiviral pseudoparticles were added to A549-hACE2+TMPRSS2 cells and luciferase reporter activity or cell binding were assessed. The background was subtracted from all data points. The data obtained were normalised with 0% luciferase activity defined as the mean of the relative luminescence units recorded from the control sample (Cells + lentiviral pseudoparticles). Data are shown as the normalised mean of three independent experiments done in triplicates ± SEM. Significance of FP-treated cells (with and without anti-FP antibodies) compared to the control (cells+ viral pseudoparticles) was determined using the two-way ANOVA test (***p < 0.001). Additionally, the significance of FP treated cells (with anti-FP antibodies) with respect to cells treated only with FP (cells+ viral pseudoparticles + FP) (###p < 0.001) (n = 3).

A cell binding assay was conducted to evaluate the effect of FH, FP and TSR4 + 5 on SARS-CoV-2 binding to lung epithelial-like cells. A549-hACE2+TMPRSS2 cells were challenged with SARS-CoV-2 lentiviral pseudoparticles treated with FH, FP, and TSR4 + 5. Pre-treatment of SARS-CoV-2 pseudoparticles with FH decreased viral binding by ~35% (**Figure 3A**). In contrast, compared to the control, FP and TSR4 + 5 promoted the binding by ~30% and ~50%, (**Figures 3B, C**), respectively. Anti-FP antibody treatment mitigated the effect of FP on viral entry and binding by ~98% and ~85% (**Figures 4A, B**), respectively. These results imply that FH and FP modulate SARS-CoV-2 viral binding, viral entrance, and subsequent infection to lung epithelial-like cells in an antagonistic and complement activation-independent manner. Furthermore, sequestering or neutralising FP limited viral binding and entry in SARS-CoV-2 infection, implicating the potential of anti-FP antibodies to limit the severity of the disease (**Supplementary Figures S4**, **S5**).

### SARS-CoV-2 infection-associated inflammation can be attenuated by FH but promoted by FP

**Figure 5:**
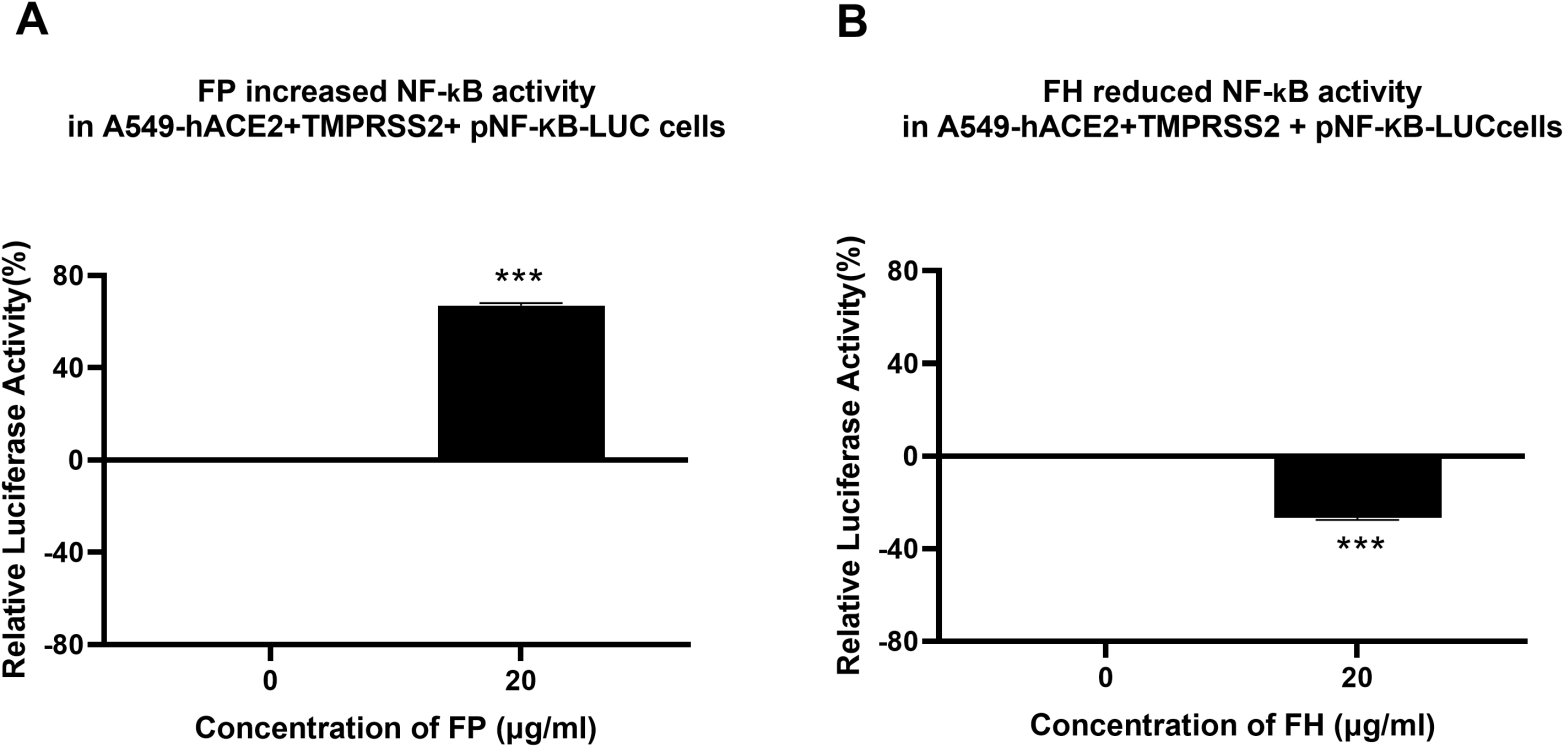
FP increases while FH reduces NF-κB activation in SARS-CoV-2- S protein challenged A549-hACE2-TMPRSS2 cells. SARS-CoV-2 S pre-treatment with FP **(A)** or FH **(B)** altered NF-kB activation. To examine the immunological role of FP and FH on NF-κB activation, A549-hACE2+TMPRSS2 cells transfected with pNF-κB-LUC were challenged with SARS-CoV-2 S protein (500ng/ml) after pre-treatment with FP or FH (20μg/ml). The cells were then incubated for 24h and examined for luciferase reporter activity. The background was subtracted from all data points. The data obtained were normalised with 0% luciferase activity defined as the mean of the relative luminescence units recorded from the control sample (A549-hACE2+TMPRSS2 cells + SARS-CoV-2 S protein). Data are demonstrated as the normalised mean of three independent experiments done in triplicates ± SEM. Significance was determined using the two-way ANOVA test (***p < 0.001) (n = 3).

Since the NF-κB pathway is often associated with pro-inflammatory signals and responses, the effect of FP or FH treatment on NF-κB activation in lung epithelial-like cells challenged with SARS-CoV-2 was evaluated using NF-κB luciferase reporter assay. FP pre-treated SARS-CoV-2 S protein exhibited a ~60% increase in NF-κB activation in A549-hACE2+ TMPRSS2 cells, while FH showed ~25% reduction in NF-κB activation compared to the control (**Figures 5A, B**), respectively.

**Figure 6:**
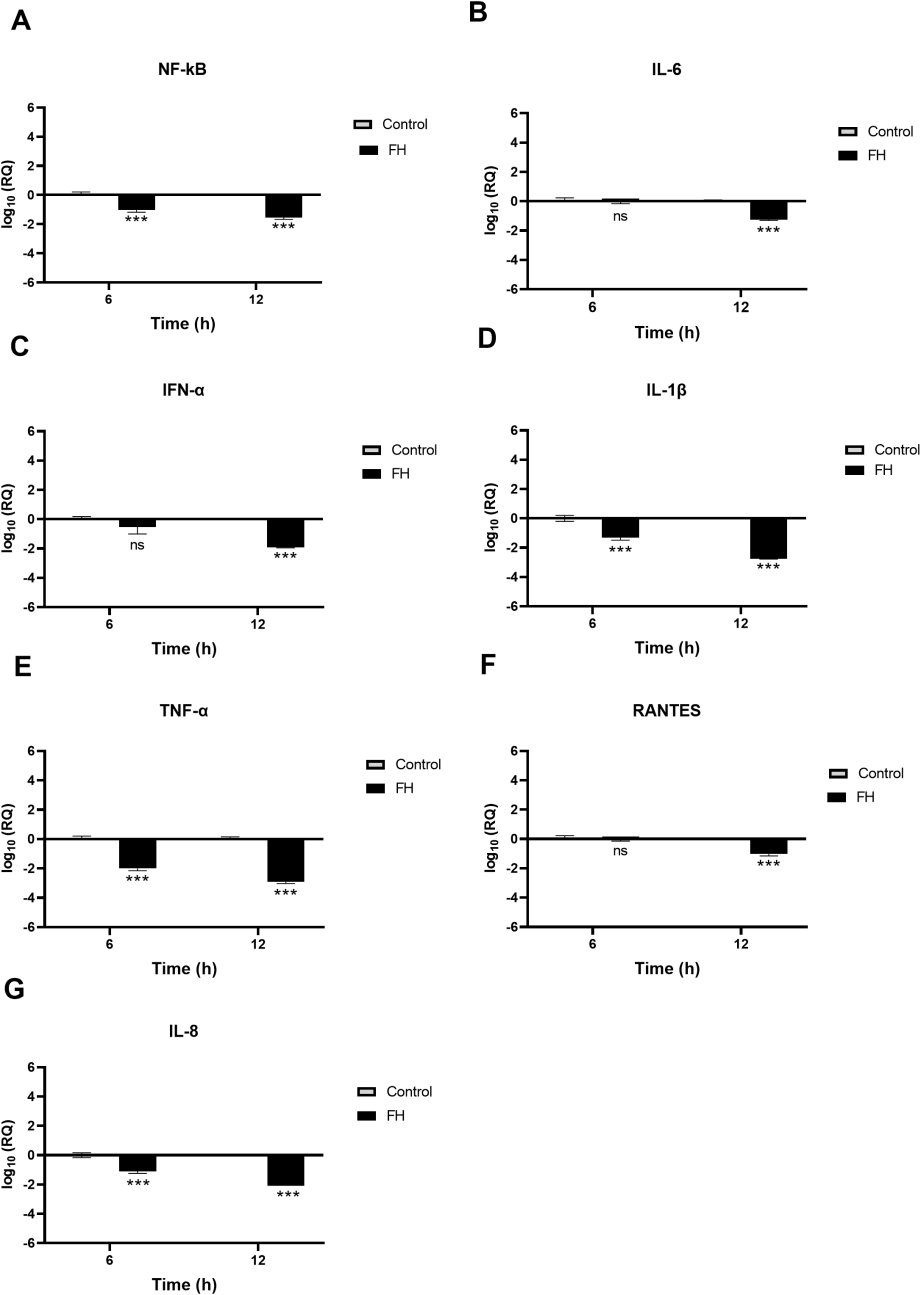
SARS-CoV-2 associated inflammation in A549-hACE2+TMPRSS2 cells is attenuated by FH. A549-hACE2+TMPRSS2 cells were challenged with and without FH (20μg/ml) pre-treated SARS-CoV-2 alphaviral pseudoparticles. Cytokines and chemokines mRNA levels were assessed using RT-qPCR for NF-κB **(A)**, IL-6 **(B)**, IFN-α **(C)**, IL-1β **(D)**, TNF-α **(E)**, RANTES **(F)** and IL-8 **(G)**. The relative expression (RQ) was calculated using the untreated cells (A549-hACE2+TMPRSS2 cells + SARS-CoV-2 alphaviral pseudoparticles) as the calibrator. The RQ value was calculated using the formula: RQ= 2-ΔΔCt. Experiments were carried out in triplicates, and error bars represent ± SEM (n =3); Significance was determined using the two-way ANOVA test (***p<0.001; ns no significance).

**Figure 7:**
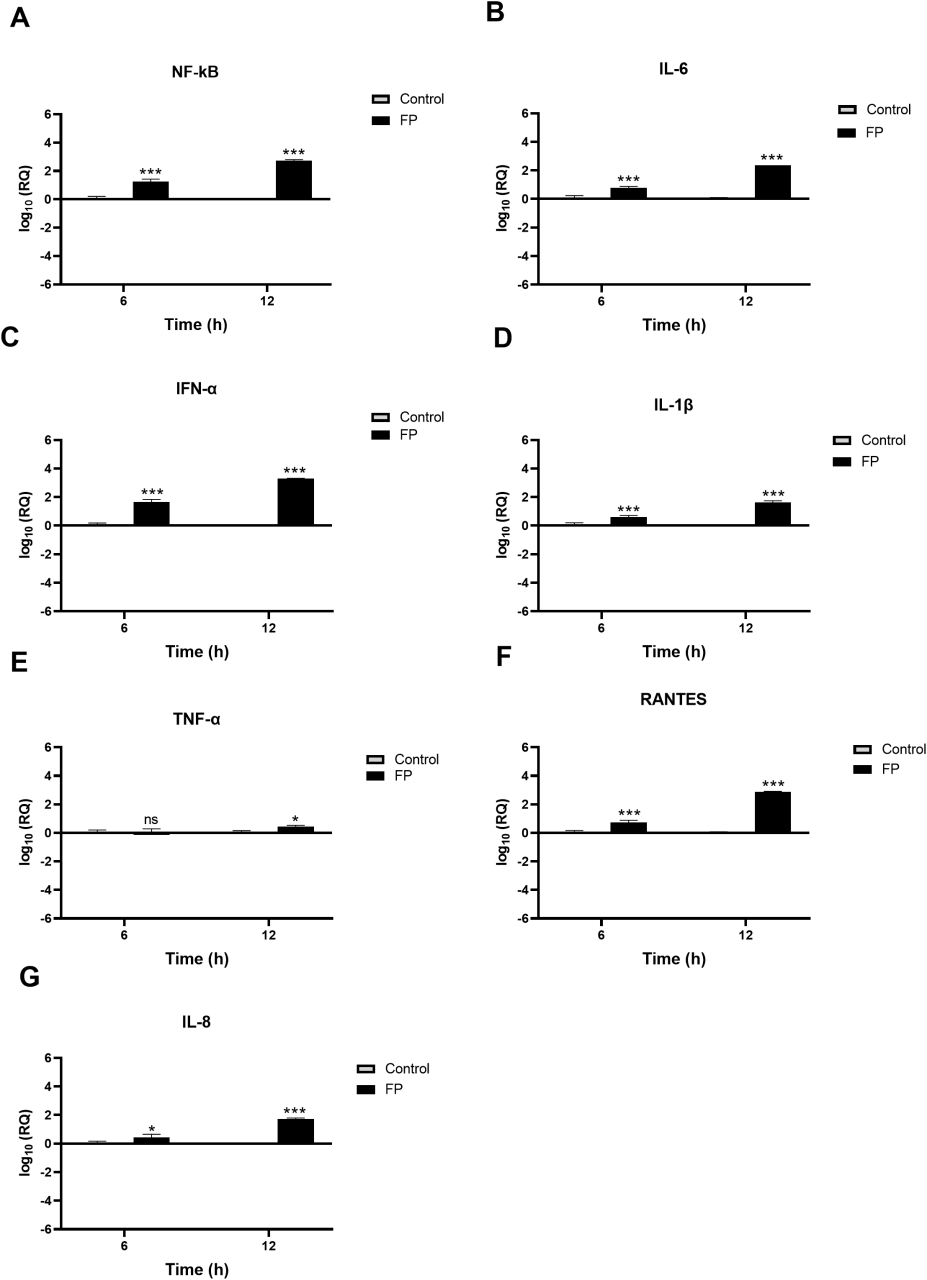
SARS-CoV-2 associated inflammation in A549-hACE2+TMPRSS2 cells is promoted by FP. FP pre-treated SARS-CoV-2 alphaviral pseudoparticles induces a pro-inflammatory response at 6h and 12h post infection in A549-hACE2 +TMPRSS2 cells. mRNA expression levels of targeted cytokines and chemokines of NF-κB **(A)**, IL-6 **(B)**, IFN-α **(C)**, IL-1β **(D)**, TNF-α **(E)**, RANTES **(F)** and IL-8 **(G)** were measured using RT-qPCR. The data were normalised through 18S rRNA expression as an endogenous control. The relative expression (RQ) was calculated using the untreated cells (A549-hACE2+TMPRSS2 cells + SARS-CoV-2 alphaviral pseudoparticles) as the calibrator. The RQ value was calculated using the formula: RQ= 2-ΔΔCt. Assays were conducted in triplicates, and error bars represent ± SEM. Significance was determined using the two-way ANOVA test (*p 0.05, ***p<0.001 and ns no significance) (n = 3).

**Figure 8:**
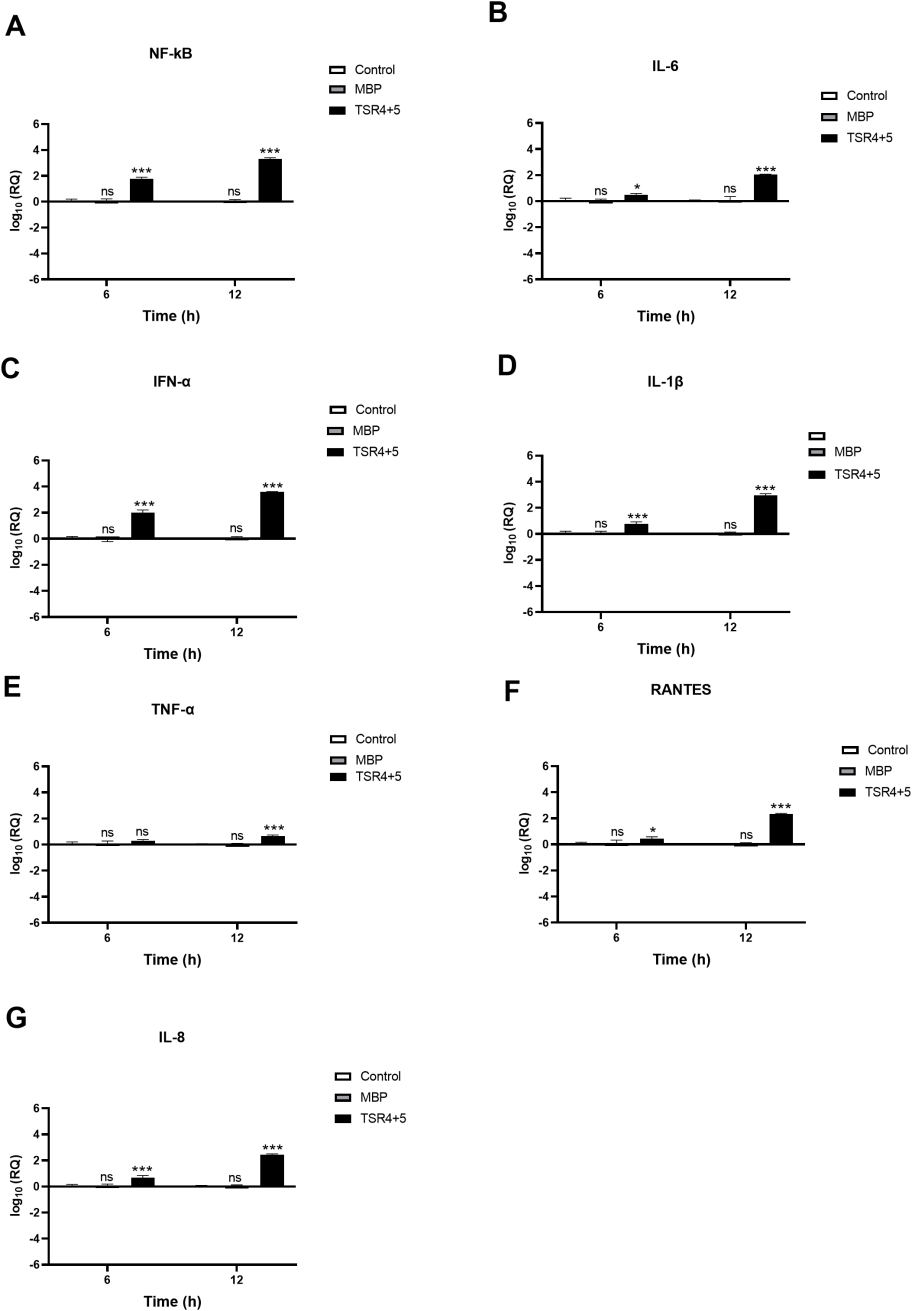
SARS-CoV-2 infection of A549-hACE2+TMPRSS2 cells induced greater pro-inflammatory response in the presence of TSR4 + 5. Pro-inflammatory events were triggered in response to TSR4 + 5 pre-treated SARS-CoV-2 alphaviral pseudoparticles in A549-hACE2 + TMPRSS2 cells at 6h and 12h post infection. The levels of gene expression of the cytokine and chemokines production were measured using qRT-PCR for NK-κB **(A)**, IL-6 **(B)**, IFN-α **(C)**, IL-1β **(D)**, TNF-α **(E)**, RANTES **(F)** and IL-8 **(G)**. The data were normalised against 18S rRNA expression as a control. Experiments were conducted in triplicates, and error bars represent ± SEM. The relative expression (RQ) was calculated using A549-hACE2 + TMPRSS2 cells + SARS-CoV-2 alphaviral pseudoparticles untreated with TSR4 + 5 as the calibrator. RQ = 2-ΔΔCt was used to calculate the RQ value. Significance was determined using the two-way ANOVA test (*p 0.05, ***p<0.001 and ns no significance) (n = 3).

Using qRT-PCR assay, we examined if FH, FP and TSR4 + 5 modulated cytokine response during SARS-CoV-2 infection. This was done by comparing the mRNA levels of pro-inflammatory cytokines and chemokines in lung epithelial-like cells challenged with SARS-CoV-2 alphaviral pseudoparticles pre-treated with 20µg/ml FH, FP or TSR4 + 5, with their corresponding controls. The results showed that FH, FP and TSR4 + 5 modulated inflammatory immune response differentially in A549-hACE2+TMPRSS2 cells (**Figures 6**–**8**), respectively. mRNA levels of pro-inflammatory cytokines such as IFN-α, IL-6, RANTES, IL-1β, IL-8 and TNF-α (and NF-κB) were downregulated in A549-hACE2+ TMPRSS2 cells challenged with SARS-CoV-2 alphaviral pseudoparticles pre-treated with FH (FH-treated cells) compared to control cells (**Figure 6**).

NF-κB gene expression levels decreased in FH-treated cells at 6h [~ -0.9 log10], peaking at 12h [-1.7 log10] (**Figure 6A**). FH-treated cells at 6h exhibited a reduction in the gene expression levels of IL-1β [~ -1.3 log10] (**Figure 6D**), TNF-α [~ -2.0 log10] (**Figure 6E**), and IL-8 [~ -0.1 log10] (**Figure 6G**) compared to their respective controls. Similarly, at 12h post-infection, FH-treated cells showed decreased mRNA levels of IL-6 [~ -1.2 log10] (**Figure 6B**), IFN-α [~ -1.9 log10] (**Figure 6C**)), IL-1β [~ -2.7 log10] (**Figure 6D**), and IFN-α [~ -2.9 log10], RANTES [~ -0.8 log10] (**Figure 6F**), and IL-8 [~ -2.1 log10] (**Figure 6G**) compared to untreated cells (**Figure 6E**). There were no significant changes in the mRNA levels of IL-6 at 6h (**Figure 6B**), IFN-α (**Figure 6C**), and RANTES (**Figure 6F**) in FH-treated cells as compared to their controls.

A549-hACE2+TMPRSS2 cells, challenged with SARS-CoV-2 alphaviral pseudoparticles that were pre-treated with FP or TSR4 + 5, the pro-inflammatory immune response was found to be upregulated (**Figures 7**, **8**). NF-κB gene expression level in FP-treated cells was increased [~ 0.7 log10] at 6h than in control cells, and it was even higher [~2.8 log10] at 12h (**Figure 7A**). At 6h, IL-6 [~0.7 log10] (**Figure 7B**), IFN-α [~1.5 log10] (**Figure 7C**), IL-1β [~0.5 log10] (**Figure 7D**), RANTES [~0.7 log10] (**Figure 7F**), and IL-8 [~0.3 log10] (**Figure 7G**) were found to be downregulated in FP-treated cells. At 12h, the mRNA levels of IL-6 (**Figure 7B**), IFN-α (**Figure 7C**), IL-1β (**Figure 7D**), TNF-α (**Figure 7D**), RANTES (**Figure 7F**), and IL-8 (**Figure 7G**) in FP - treated cells, were even more upregulated [~2.7 log10, ~3.4 log10, ~1.5 log10, ~0.2 log10, ~3.0 log10, and ~1.4 log10, respectively]. There was no significant alteration in the mRNA levels of IFN-α at 6h in FP-treated cells respective to the control (**Figure 7E**).

Similarly, TSR4 + 5 - treated cells showed elevated NF-κB gene expression levels (~0.2 log10) than control cells, with even higher levels at 12h [~2.0 log10] (**Figure 8A**). There was no significant difference between the control and the MBP-treated cells (TSR4 + 5 fusion protein) (**Figure 8**). Compared to their respective controls, at 6h, the mRNA levels of IL-6 [~0.5 log10] (**Figure 8B**), IFN-α [~1.9 log10] (**Figure 8C**), IL-1β [~0.7 log10] (**Figure 8D**), TNF-α [~0.1 log10] (**Figure 8E**), RANTES [~0.2 log10] (**Figure 8F**), and IL-8 [~0.8 log10] (**Figure 8G**) in TSR4 + 5-treated cells were augmented as compared to the control. The downregulation in mRNA levels of IL-6 [~ 2.2 log10] (**Figure 8B**), IFN-α [~3.7 log10] (**Figure 8C**), IL-1β [~2.7 log10] (**Figure 8D**), TNF-α [~0.6 log10] (**Figure 8E**), RANTES [~2.3 log10] (**Figure 8F**), and IL-8 [~2.2 log10] (**Figure 8G**), were considerably more significant at 12h in TSR4 + 5- treated cells as compared to the untreated control. These findings suggest that FP and FH differentially modulate NF-κB activation and associated inflammatory response in SARS-CoV-2 infection.

### FP interacts with spike and ACE2 in a tripartite complex

**Figure 9:**
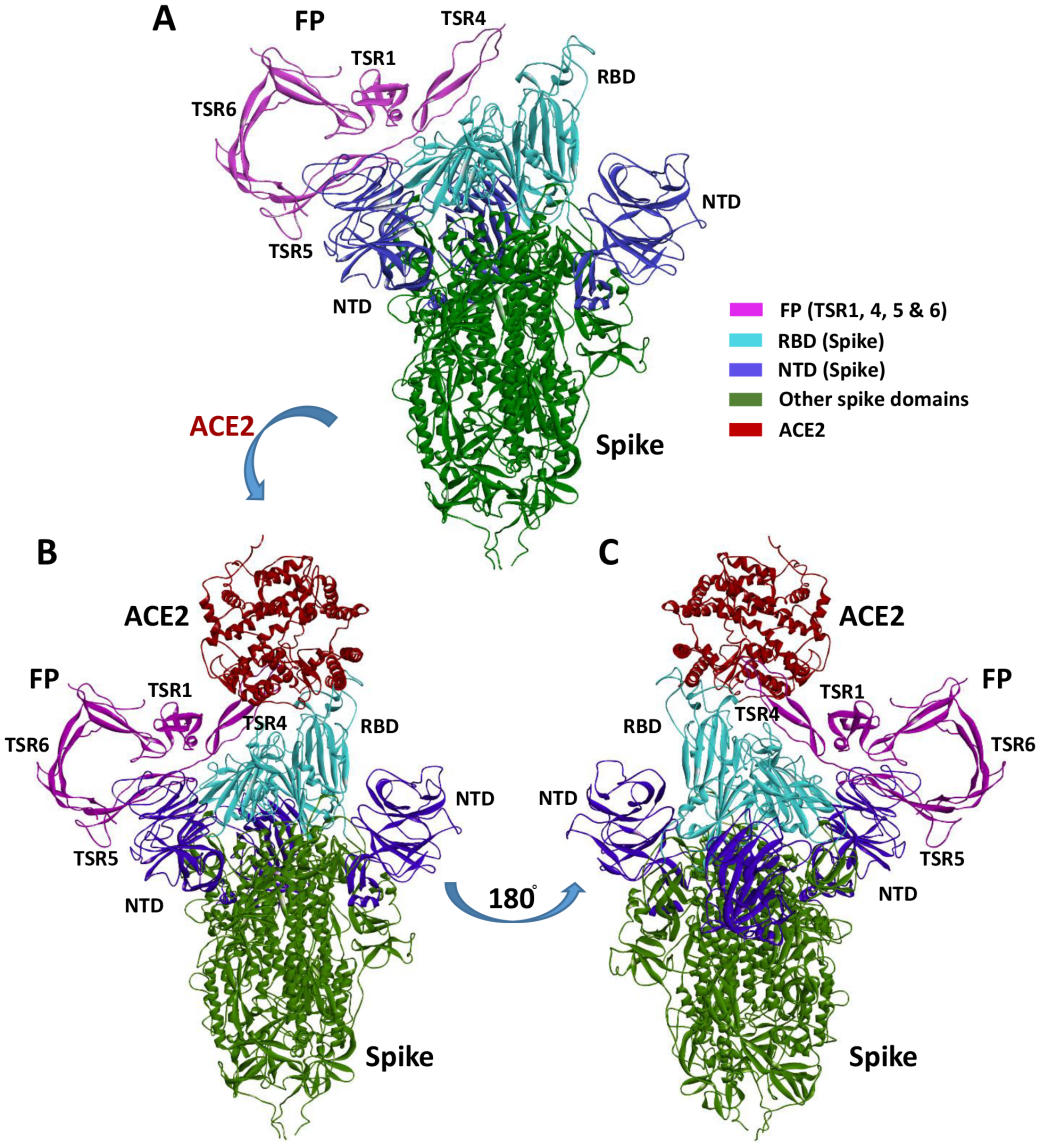
Cartoon representation of FP interaction with spike and ACE2. **(A)** Interaction of FP with spike RBD and NTD through TSR4 and TSR5 domains. **(B, C)** A tripartite complex representation of FP, spike and ACE2.

A blind docking approach was attempted to generate a complex of FP and S protein. The second ranked docked pose concurred with the *in vitro* observation that TSR4 and 5 domains of FP interacted with RBD and NTD domains of S protein, respectively through H-bonding, electrostatic and hydrophobic interactions (**Table 2**; **Figure 9A**). A tripartite complex structure of FP, S and ACE2 was created by docking electron microscopy structure of ACE2 to FP bound spike protein. In the first ranked pose, ACE2 was found to interact with S as reported in the electron microscopy structure (PDB ID: 7KNB). It was observed that FP interacted with both S and ACE2 in the tripartite complex through various non-bonded contacts in each subunit (**Table 2**; **Figures 9B, C**). In this structure, TSR4 domain of FP was also found proximal to ACE2 receptor and showed interaction with both S and ACE2. The binding affinity of ACE2 receptor with unbound and FP-bound S proteins was compared through Zdock score and binding free energy. These scores indicated that ACE2 receptor had a strong affinity for FP-bound S as compared to unbound S protein (**Table 3**). This suggests that FP may enhance the affinity of S for ACE2 by interacting with both proteins through a tripartite complex. 

**Table 2:** Interaction details of docked complexes of FP, Spike and ACE2.

Receptor	Ligand	H-bonding residues		Electrostatic interaction residues		Hydrophobic interaction residues	
		Receptor	Ligand	Receptor	Ligand	Receptor	Ligand
Spike	FP	ASN122, ASN125, TYR145, THR345, LYS356, LYS444	CYS269,GLN281, ASP356, GLN363, GLN364, GLN365, ASN428	PHE157	ASP366	LYS129, CYS131, CYS166, THR167, PHE168, ALA344, LEU441	PRO268, VAL310, HIS358, ARG359, ALA361
Spike	ACE2	TYR473, ALA475, GLN493, GLY496, GLN498, THR500, ASN501, GLY502	SER19, GLU23, ASP38, ASN330, LYS353, GLY354, ASP355	GLU484	LYS31	TYR453, LEU455, PHE486, TYR489, TYR505	LYS31, HIS34, MET82, LYS353
ACE2	FP	GLU564	HIS291	TYR215	SER255	LEU91, PRO565	HIS291

**Table 3:** Comparative docking score and binding free energy of docked complexes of FP, Spike and ACE2.

Complex	Zdock score	Binding free energy (kcal/mol)
Spike & FP	21.52	-3867.90
Spike & ACE2	16.40	-1315.89
Spike-FP & ACE2	18.58	-1653.68

## Discussion

The COVID-19 pandemic has resulted in the loss of millions of lives and continues to pose significant financial burdens with rise of novel superspreading mutants having immune-evasive potential (27). Therefore, gaining a comprehensive understanding of the pathogenesis of SARS-CoV-2 remains crucial for a better managing the infection and preparing for future outbreaks. Extensive evidence emerged during the pandemic that highlighted the involvement of the complement system, particularly the dysregulation of the alternative pathway in COVID-19 pathogenesis and its immunopathological consequences (11, 28, 29). Additionally, it has been observed that the S protein of SARS-CoV-2 can directly activate the alternative pathway (13). Out of the two key regulators of the alternative pathway, a reduction of FH levels and elevated levels of FP in severe SARS-CoV-2 infection and in those with fatal outcomes have been reported (30, 31). However, the role of FH and FP in initial immune response against SARS-CoV-2 infection independently of complement activation, remains unexplored. Thus, in this study, we investigated FH and FP immune functions in SARS-CoV-2 inflection. This study demonstrates that FH and FP, acting as soluble PRRs of the innate immunity, can directly interact with both SARS-CoV-2 S and RBD proteins and modulate the SARS-CoV-2 infectivity. A549 cells expressing human ACE2 and TMPRSS2 (A549-hACE2+TMPRSS2 cells) were used as a relevant *in vitro* respiratory epithelium model (10). SARS-CoV-2 viral pseudoparticles were utilised as a safe model to study SARS-CoV-2 infection (10).

SARS-CoV-2, a highly pathogenic virus, requires reliable assays to study the interactions between the virus and its host. Studies involving the live virus must adhere to stringent biosafety level (BSL) protocols in specialised laboratories (32). The production and application of SARS-CoV-2 S lentiviral and alphaviral pseudoparticles provide valuable tools for various research purposes. These include studying virus-host dynamics, characterising neutralising antibodies, and developing inhibitors in the form of small molecules (33).

FH serves as a critical negative regulator in the complement alternative pathway (6). Notably, lung fibroblasts have been found to secrete FH locally, emphasising its role in lung homeostasis and immune regulation (34). On the other hand, FP acts as a positive regulator of the alternative pathway, with infiltrating neutrophils being a primary local source of FP secretion via secretory granules within the lung (7). Interestingly, both FH and FP have been shown to bind and inhibit IAV cell entry, their inhibitory effects on IAV infection are dependent on the specific IAV subtype (6, 7). Additionally, FH and FP have been demonstrated to attenuate inflammatory responses in A549 cells, further highlighting their immunomodulatory properties (6, 7).

The FP monomer consists of seven thrombospondin type I repeats (TSR). TSR4 and 5 and particularly critical for binding to C3bBb, indicating that these TSRs play a vital role in stabilising the C3 convertase complex (23). Interestingly, a recent study showed that recombinant TSR4 + 5 protein binds to *M. bovis* BCG, indicating its function as a soluble pattern recognition receptor (PRR) (24). The study also found that TSR4 + 5 inhibits the uptake of *M. bovis* BCG by macrophages during phagocytosis, which affects both pro-inflammatory and anti-inflammatory cytokine responses. These findings highlight the significant role TSR4 + 5 have in mediating pathogenic interactions.

In contrast to most complement proteins which are produced by hepatocytes, properdin is primarily synthesised and secreted by leukocytes, including T cells, monocytes, alveolar macrophages, dendritic cells, and granulocytes (35). Additionally, expression of properdin mRNA has been detected in airway epithelial cells (35). Notably, neutrophils contain significant amounts of properdin in their secondary granules (35). In states of neutropenia, properdin levels in the serum decrease by approximately 19–32% (35). It has been reported that neutrophils are the primary local source of properdin secretion within the lungs (7). Dysregulation of properdin levels have been associated with severe tissue damage and, in some cases, persistent chronic inflammation (36). For example, elevated mRNA levels of properdin have been detected in patients with asthma.

Cell binding assay showed that FH significantly reduced lentiviral pseudoparticle binding to and infection of A549-hACE2+TMPRSS2 cells, whereas FP and TSR4 + 5 enhanced cell binding and infectivity. Thus, we conducted *in-silico* studies, which revealed that FP interacted with S protein through TSR4 + 5 domains, forming a tripartite complex. This complex exhibited a high binding affinity to the host ACE2 receptor via TSR4, leading to an increased affinity between the virus and host ACE2 compared to the virus without FP. These observations suggest a possible mechanism by which FP enhances virus infection and replication, resulting in the poor prognosis observed in infected individuals. Luciferase reporter assay revealed that pre-treatment of SARS-CoV-2 lentiviral pseudoparticles with FH significantly reduced viral transduction within A549-hACE2+TMPRSS2 cells. Conversely, treatment with FP or TSR4 + 5 resulted in an increase in viral transduction compared to the control. Therefore, FH is a cell entry inhibitor for the SARS-CoV-2, whilst FP is a viral entry enhancer into lung epithelial-like cells. Rabbit anti-human-FP polyclonal antibodies seem to reverse FP-mediated enhancement of viral cell entry and binding. Furthermore, we included a rabbit anti-human-SP-D polyclonal antibody as a negative antibody control. Anti-SP-D does not recognise any FP or SARS-CoV-2 components and had no effect on viral binding or transduction, thereby confirming that the observed reversal by anti-FP antibodies was not due to non-specific antibody effects.

Neutrophils serve as a reservoir for properdin and can release it rapidly via secretory granules upon activation (37). This local release of properdin from neutrophils is believed to be the primary factor influencing alternative pathway activity, as plasma properdin levels are typically low (37). Notably, neutrophils have been implicated in driving inflammatory responses during SARS-CoV-2 infection (38). The findings suggest that sequestering excessive FP may have the potential for an effective therapeutic strategy in limiting immunopathogenesis for severe SARS-CoV-2 infection.

Dysregulation of inflammatory response is one of the primary drivers in the transition of SARS-CoV-2 infection to moderate/severe COVID-19 (39, 40). Elevated serum levels of IL-1β, IL-6, IL-8, and TNF-α have been observed in SARS-CoV-2 infection, and are associated with the severity of the disease (39, 40). High levels of IL-6, TNF-α, MIP-2 and IL-8 gene expression have been found in alveolar type II cells challenged with SARS-CoV-2 (41). Furthermore, both mild and severe SARS-CoV-2 infections have been characterised by dysregulation of NF-κB activities (42–44). NF-κB signalling is vital for an effective immune response toward viral infection (45). Nevertheless, dysregulation of NF-κB functions is central to SARS-CoV-2 immunopathogenesis (42), because it has been associated with high levels of gene expression and proteins of pro-inflammatory mediators, including IL-1, IL-2, IL-6, IL-12, TNF-α, IL-8, MIP-1, MCP1 and RANTES in severe COVID-19 (42, 46–48), and hence, its therapeutic significance (49, 50). In this study, the gene expression levels of NF-κB were downregulated in A549-hACE2+TMPRSS2 cells challenged with FH-treated SARS-CoV-2 alphaviral pseudoparticles (FH-treated cells). In contrast, FP or TSR4 + 5 upregulated the NF-κB transcript expression. We also found a decrease in NF-κB activation in A549-hACE2+TMPRSS2 cells challenged with FH treated SARS-CoV-2 S protein, whist there was an increase in NF-κB activation in FP-treated cells. Thus, FH may reduce hyperinflammation via the downregulation of NF-κB. On the contrary, FP can be exacerbating pro-inflammatory response in SARS-CoV-2 infection in the pulmonary tissues.

Another pro-inflammatory key player in the inflammatory response to viral infection is IL-1β (51). SARS-CoV-2 influences the activation of IL-1β, which can subsequently affect IL-6 and TNF-α (52, 53). SARS-CoV-2-induced cytokine storm is facilitated by IL-1β (54). Elevated levels of IL-1β in the peripheral blood and bronchoalveolar lavage fluid have been observed in patients with severe SARS-CoV-2 infection (55, 56). IL-1ß targeted therapy prevents SARS-CoV-2 induced cell death (57). Also, IL-1 receptor blocking in the early stage of the disease was found to be an effective treatment against respiratory failure, cytokine storm development, and hyperinflammation in COVID-19 patients (58). In this study, we observed that FH-treated cells demonstrated a reduction in the gene expression levels of IL-1β, while they were higher in FP and TSR4 + 5 treated cells. Elevated serum levels of TNF-α have been observed in severe SARS-CoV-2 infection (59, 60). TNF-α is a crucial immune player in limiting viral infections (61, 62). However, high levels of TNF-α contribute to lung damage and poor outcomes in severe COVID-19 patients (60). Combination of anti-TNF-α and anti-IFN-γ therapy in severe SARS-CoV-2 infection was able to reduce tissue damage and mortality (63). The mRNA levels of TNF-α in FH-treated cells were lower, whereas they were higher in FP and TSR4 + 5 treated cells. The findings imply FH could reduce TNF-α associated complications in SARS-CoV-2 infection, whilst FP may promote them.

IL-6 serum levels were higher in individuals with SARS-CoV-2-associated pneumonia, linked to the disease severity and mortality (64). Elevated levels of IL-6 are associated with a poor prognosis due to its contribution to inflammation and cytokine storm (64). Interestingly, COVID-19 patients, who are at risk of suffering cytokine storm, seem to respond well to tocilizumab, a monoclonal antibody that targets IL-6 receptors (64). Decreased mRNA levels of IL-6 by FH whilst its elevation in FP or TSR4 + 5 treated cells appear to suggest that FH might prevent the transition of SARS-CoV-2 infection to the severe form of the disease through limiting dysregulation of IL-6 levels, whereas FP may contribute to promoting IL-6 abnormality.

Type I IFN (IFN-α & IFN-β) is a major cytokine in localising and preventing viral infection via inducing the expression of interferon-stimulated genes (ISGs) (65). Elevated IFN-type 1 (IFN-1) levels could contribute to hyperinflammation in the progression to severe SARS-CoV-2 infection via various mechanisms (65). However, early studies have shown limited IFN-1 response in SARS-CoV-2 infection (65). Recent reports on the role of IFN-1 in the development of severe SARS-CoV-2 infection have surfaced (65). Also, a retrospective study revealed that the administration of IFN-α early reduced mortality, whereas its use in severe infection increased mortality and delayed recovery (66). Here, we showed that FH-treated cells exhibited a reduction in IFN-α gene expression levels, while in both FP and TSR4 + 5 treated cells, an elevation was observed with respect to the controls. Thus, FH could contribute to the immune regulatory mechanism in preventing unnecessary inflammation by limiting the action of IFN-α in immunopathogenesis in disease progression. At the same time, FP may be a co-factor in the disease pathology.

In individuals with severe SARS-CoV-2 infection, IL-8 is associated with significant neutrophil infiltration, respiratory failure, and acute kidney damage (67). IL-8 is a potent pro-inflammatory cytokine crucial for activating and recruiting neutrophil cells during inflammation (67). Severe SARS-CoV-2 patients are more likely to experience neutrophilia than mild disease patients (67). A prior anti-CXCL-8 therapy prevents the onset of severe lung injury (68). In this study, we show that FH treatment downregulated IL-8 mRNA level, whereas it was upregulated in FP and TSR4 + 5 treated cells. These findings suggest the possible function of FH in preventing lung injury by inhibiting IL-8 in SARS-CoV-2 infection, while FP may exacerbate it in a complement activation-independent manner. Interestingly, various studies have demonstrated that high levels of neutrophil infiltration in severe SARS-CoV-2 infection is correlated with poor clinical outcomes (69). Since it is known that neutrophils release properdin from specific granules (7), FP may promote a feedback loop in which more IL-8 is produced in SARS-CoV-2 infection, leading to more neutrophil infiltration. That can result in elevated levels of FP being secreted, worsening the infection.

Lastly, elevated serum levels of RANTES were observed in mild and severe SARS-CoV-2 patients with respect to healthy control (70). RANTES (CCL5) is a potent leucocyte chemoattractant that induces the migration of various immune cells, including T cells, natural killer cells, dendritic cells, monocytes, basophils, and eosinophils (71, 72). High level of RANTES is associated with acute renal failure and liver damage in individuals with severe COVID-19 (73). Targeting RANTES early on in viral infection may improve viral clearance and help localise the viral infection (31). In this study, we found that FH-treated cells exhibited reduced gene expression levels of RANTES, whereas mRNA levels of RANTES were elevated in FP and TSR4 + 5 treated cell compared to their respective controls. These results suggest an immune role for FH in limiting viral infection and enhancing viral clearance via the involvement via RANTES in SARS-CoV-2 infection, while FP may delay viral clearance.

A high level of FP in severe COVID-19 patients (29) may be explained by its role in promoting both infection and inflammation resulting in the disease severity. Besides, the insufficient levels of FH in severe SARS-CoV-2 may contribute to the disease progression. While this study provides valuable insights into the potential immune protective role of FH and the immunopathological role of FP in COVID-19, additional experiments using clinical isolates from different variants and lineages are essential to understand the infection dynamics comprehensively. Moreover, *in vivo*, studies are required to evaluate the impact of local FH and FP in the lung microenvironment and explore combination therapies to mitigate complications associated with SARS-CoV-2 infection.

This study acknowledges few limitations. First, the use of pseudotyped SARS-CoV-2 particles, while a safe and widely accepted model for studying viral entry and immune interactions, does not fully capture the complexity of live virus infection. Although spike-only pseudoparticles were sufficient for assessing binding interactions, our use of pseudoparticles expressing all four structural proteins (S, E, M, and N) for cell uptake and cytokine response was aimed at better mimicking the immunopathogenic features of SARS-CoV-2. Nonetheless, the absence of other viral components and accessory proteins may be a limiting component. Second, the *in vitro* experiments, conducted in a lung epithelial-like cell line (A549-hACE2+TMPRSS2), may not entirely reflect *in vivo* conditions, particularly regarding tissue microenvironment, immune cell interactions, and systemic inflammation. Third, our qPCR profiling was limited to 6h and 12h time points, which may have missed early transcriptional events (e.g., at 3h) or later regulatory phases (e.g., at 24h). Including these additional time points in future studies could provide a more comprehensive view of the temporal dynamics of cytokine responses. Fourth, while our findings indicate distinct roles for FH and FP in modulating SARS-CoV-2 infection and inflammation, these effects may be influenced by additional cofactors or receptors not examined in this study. Future work employing live SARS-CoV-2 in animal models or primary human cells is essential to validate these findings and explore the broader immunological context of complement protein interactions during infection. Lastly, while *in silico* analysis provides valuable insights into molecular structures and interactions, its reliability is limited by the accuracy of the 3D structural models used. The findings of this study suggest that FP may facilitate the interaction between the SARS-CoV-2 S protein and host ACE2 by binding to both. However, the absence of a suitable full-length 3D model of FH prevented a computational evaluation of its potential interactions with these proteins.

**Figure 10:**
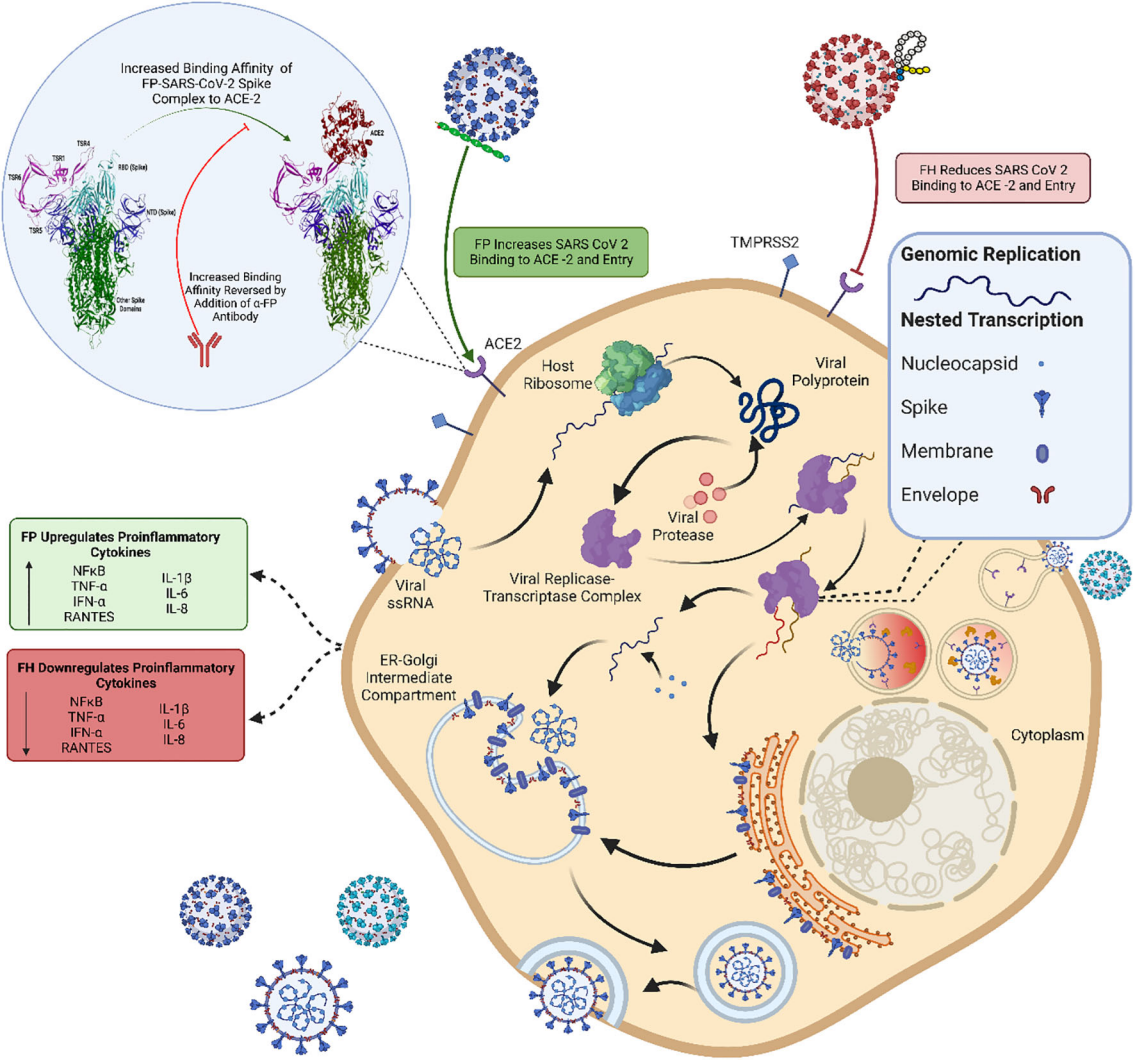
Immune effector function of FP and FH in SARS-CoV-2 infection independent of complement activation. The SARS-CoV-2 virus binds to host cells through the ACE2 receptor using its S protein. After fusion with the cell membrane, the viral RNA is released into the host cytoplasm, initiating viral replication, protein synthesis and viral release into the extracellular environment contributing to the spread of SARS-CoV-2 infection. In this study, FP and FH were investigated separately to understand their effects on SARS-CoV-2 infection without activating the complement cascade. FP was found to enhance the binding affinity between the SARS-CoV-2 S protein and ACE2, leading to increased viral entry into host cells and subsequent infection. This increased viral load triggered the upregulation of proinflammatory cytokines, contributing to the inflammatory response. Conversely, FH was observed to reduce the binding and entry of SARS-CoV-2 into host cells. Consequently, the lower viral entry mediated by FH resulted in decreased production of proinflammatory cytokines, potentially mitigating the immune response. These findings shed light on the immunomodulatory role of FH and the immunopathological role of FP in COVID-19. Moreover, they provide insights into the potential association between elevated levels of properdin and insufficient levels of FH observed in severe COVID-19 patients. This figure was modelled based on a published figure with permission of the rights holder, Elsevier GmbH, from Varghese et al., 2020 (74). Created with BioRender.com.

In summary, this study has unveiled a novel finding that FH and FP can interact with the RBD of the SARS-CoV-2 S protein in a complement- activation independent manner (**Figure 10**). This interaction of FH hinders the virus from binding to its cell surface receptors, resulting in a reduction in SARS-CoV-2 infection of A549 cells that express both human ACE2 and TMPRSS2, while FP enhances the viral infection. Furthermore, the presence of FH led to a decrease in the expression levels of proinflammatory cytokines and chemokines, including IL-1β, IL-8, IL-6, TNF-α, IFN-α, NF-κB, and RANTES whereas these pro-inflammatory mediators were increased in the case of FP. Thus, complement proteins can act as a direct protective mechanism against viral infections; however, they, as is the case with FP, may also contribute to the disease severity independent of their roles in complement activation. This study sheds light on a mechanism through which our innate immune system offers protection as well as plays a part in SARS-CoV-2 immunopathology (**Figure 10**).

## Data Availability

The original contributions presented in the study are included in the article/**Supplementary Material**, further inquiries can be directed to the corresponding author/s.

## Supplementary material

Supplementary Figure 1 Characterisation of purified complement proteins. The immunoreactivity of the purified complement proteins was assessed using western blotting. A PVDF membrane was employed and probed at RT for 1h with specific antibodies (1:1,000 dilution). The antibodies used were rabbit-anti-human Properdin polyclonal antibodies, and mouse-anti-human FH monoclonal antibody (MRCOX23). Additionally, a protein ladder spanning a range of 250 to 10 kDa was included. Following the primary antibody incubation, the membrane was subsequently incubated with secondary antibodies, either goat anti-rabbit IgG HRP-conjugate or goat anti-mouse IgG HRP-conjugate (1:1,000 dilution), for 1h at RT. The resulting bands corresponding to the respective proteins were observed. For FH was observed at ~155 kDa (A), Properdin exhibited a band ~55 kDa (B) and TSR4 + 5 was at ~55 kDa (C), after developing the colour using 3,3′-diaminobenzidine (DAB) substrate.

Supplementary Figure 2 SARS-CoV-2 interacted with TSR4 + 5 via its S Protein RBD. TSR4 + 5 bound both SARS-CoV-2 S and RBD proteins in a dose-dependent manner. Decreasing concentration of immobilised TSR4 + 5 (1, 0.5, 1.25, and 0 μg/well) were coated in a 96-well plate using Carbonate-Bicarbonate (CBC) buffer, pH 9.6 at 4°C overnight. After washing out the excess CBC buffer with PBS, three times, a constant concentration of virus proteins (1 μg/well) was added to corresponding wells, followed by incubation at 37°C for 2h. After washing out the unbound proteins, the wells were probed with rabbit anti-SARS-CoV-2 S (1:5000; 100 μ/well). MBP and BSA were used as negative control.

Supplementary Figure 3 The cartoon representation of the full-length 3D structure of FH consists of 20 short consensus/complement repeat (SCR) domains. This structure was sub-optimal due to several unfolded regions and large, extended structures that were unsuitable for docking. The structure was modelled using ModellerCartoon representation of modelled FH structure. Full-length 3D structure of FH was modelled using Modeller10.1.

Supplementary Figure 4 FH, FP and TSR 4 + 5 modulate SARS-CoV-2 pseudoparticle entry into A549-hACE2+TMPRSS2 cells. Luciferase reporter activity of FH, FP or TSR 4 + 5 (20 μg/mL) pre-treated cells transduced with SARS-CoV-2 lentiviral pseudoparticles for 24h was measured. The assay was conducted in triplicates, and error bars represent ± SEM. The statistical significance of Spike, BSA or MBP treated cells were compared to untreated cells was determined by using the two-way ANOVA (^+++^p < 0.05). Similarly, the statistical significance of FH or FP pre-treated cells challenged with SARS-CoV-2 lentiviral pseudoparticles was also determined by using the two-way ANOVA by comparing to cells that were only challenged with the pseudotypes (***p < 0.05), or to BSA pretreated cells challenged with the pseudotypes (^###^p < 0.05). Since the recombinant TSR4 + 5 is tagged with MBP, the statistical significance of TSR4 + 5 pre-treated cells challenged with the pseudotypes was assessed using the two-way ANOVA by comparing to cells that were pretreated with MBP and then challenged with the pseudotypes (^†††^p < 0.05). Notably, anti-FP has statistically significant (^&&&^p < 0.05) reduced the viral entry effect of FP treated cells, while no significantly found as compared to cells treated with Spike or BSA (ns > 0.05). The results clearly show that FH pretreatment significantly reduces viral entry, while pretreatment with either FP or TSR 4 + 5 significantly increases viral entry. Finally, no statistically significant difference using the two-way ANOVA was identified between BSA or MBP pre-treated cells when as compared to cells directly challenged with the pseudotypes (ns > 0.05).

Supplementary Figure 5 Binding of FH or PF pre- treated SARS-CoV-2 pseudoparticles to A549-hACE2+TMPRSS2 cells. SARS-CoV-2 lentiviral pseudoparticles were used to transduce A549-hACE2+TMPRSS2 cells, which were pre-incubated with FH or FP (20 µg/mL). The wells were probed with rabbit anti-SARS-CoV-2 S (1:200) polyclonal antibodies after being washed and fixed with 1% v/v PFA for 1 min. Viral entry assay was conducted in triplicates, and error bars represent ± SEM. Statistical significance was determined using the two-way ANOVA (^+++^p < 0.05) for cells treated with Spike, BSA, or MBP as compared to untreated cells. For cells pre-treated with FH or FP and then challenged with SARS-CoV-2 lentiviral pseudoparticles, significance was compared to cells only challenged with pseudotypes (***p < 0.05) or to BSA pre-treated cells subsequently challenged with pseudotypes (^###^p < 0.05). Recombinant TSR4 + 5 (tagged with MBP), the significance of TSR4 + 5 pre-treated cells challenged with pseudotypes was evaluated against cells pre-treated with MBP followed by a pseudotype challenge (^†††^p < 0.05). Importantly, anti-FP has statistically significant (^&&&^p < 0.05) reversed the binding effect of FP treated cells, whereas no significantly found when compared to cells treated with Spike or BSA (ns > 0.05). These findings revealed that FH pretreatment reduced viral binding, whereas FP or TSR 4 + 5 pretreatment enhanced it. No significant difference was observed between BSA or MBP pre-treated cells and those directly challenged with pseudotypes (ns > 0.05).

## References

[1] Hu B Guo H Zhou P Shi Z-L. Characteristics of SARS-coV-2 and COVID-19. Nat Rev Microbiol. (2021) 19:141–54. doi: 10.1038/s41579-020-00459-7, PMID: 33024307 PMC7537588

[2] Lamers MM Haagmans BL. SARS-coV-2 pathogenesis. Nat Rev Microbiol. (2022) 20:270–84. doi: 10.1038/s41579-022-00713-0, PMID: 35354968

[3] Merle NS Church SE Fremeaux-Bacchi V Roumenina LT. Complement system part I–molecular mechanisms of activation and regulation. Front Immunol. (2015) 6:262. doi: 10.3389/fimmu.2015.00262, PMID: 26082779 PMC4451739

[4] Murugaiah V Varghese PM Beirag N DeCordova S Sim RB Kishore U. Complement proteins as soluble pattern recognition receptors for pathogenic viruses. Viruses. (2021) 13:824. doi: 10.3390/v13050824, PMID: 34063241 PMC8147407

[5] Chen M Daha MR Kallenberg CG. The complement system in systemic autoimmune disease. J Autoimmun. (2010) 34:J276–J86. doi: 10.1016/j.jaut.2009.11.014, PMID: 20005073

[6] Murugaiah V Varghese PM Saleh SM Tsolaki AG Alrokayan SH Khan HA. Complement-independent Modulation of influenza A virus infection by Factor H. Front Immunol. (2020) 11:355. doi: 10.3389/fimmu.2020.00355, PMID: 32269562 PMC7109256

[7] Varghese PM Mukherjee S Al-Mohanna FA Saleh SM Almajhdi FN Beirag N. Human properdin released by infiltrating neutrophils can modulate Influenza A virus infection. Front Immunol. (2021) 12:747654. doi: 10.3389/fimmu.2021.747654, PMID: 34956182 PMC8695448

[8] Ip WE Chan KH Law HK Tso GH Kong EK Wong WH. Mannose-binding lectin in severe acute respiratory syndrome coronavirus infection. J Infect diseases. (2005) 191:1697–704. doi: 10.1086/429631, PMID: 15838797 PMC7199483

[9] Houser KV Broadbent AJ Gretebeck L Vogel L Lamirande EW Sutton T. Enhanced inflammation in New Zealand white rabbits when MERS-CoV reinfection occurs in the absence of neutralizing antibody. PloS pathogens. (2017) 13:e1006565. doi: 10.1371/journal.ppat.1006565, PMID: 28817732 PMC5574614

[10] Beirag N Varghese PM Neto MM Al Aiyan A Khan HA Qablan M. Complement activation-independent attenuation of SARS-CoV-2 infection by C1q and C4b-binding protein. Viruses. (2023) 15:1269. doi: 10.3390/v15061269, PMID: 37376569 PMC10305604

[11] Java A Apicelli AJ Liszewski MK Coler-Reilly A Atkinson JP Kim AH. The complement system in COVID-19: friend and foe? JCI Insight. (2020) 5. doi: 10.1172/jci.insight.140711, PMID: 32554923 PMC7455060

[12] Siggins MK Davies K Fellows R Thwaites RS Baillie JK Semple MG. Alternative pathway dysregulation in tissues drives sustained complement activation and predicts outcome across the disease course in COVID-19. Immunology. (2023) 168:473–92. doi: 10.1111/imm.13585, PMID: 36175370 PMC9537932

[13] Yu J Yuan X Chen H Chaturvedi S Braunstein EM Brodsky RA. Direct activation of the alternative complement pathway by SARS-CoV-2 spike proteins is blocked by factor D inhibition. Blood. (2020) 136:2080–9. doi: 10.1182/blood.2020008248, PMID: 32877502 PMC7596849

[14] Ramlall V Thangaraj PM Meydan C Foox J Butler D Kim J. Immune complement and coagulation dysfunction in adverse outcomes of SARS-CoV-2 infection. Nat Med. (2020) 26:1609–15. doi: 10.1038/s41591-020-1021-2, PMID: 32747830 PMC7809634

[15] Alosaimi B Mubarak A Hamed ME Almutairi AZ Alrashed AA AlJuryyan A. Complement anaphylatoxins and inflammatory cytokines as prognostic markers for COVID-19 severity and in-hospital mortality. Front Immunol. (2021) 12:668725. doi: 10.3389/fimmu.2021.668725, PMID: 34276659 PMC8281279

[16] Meroni PL Croci S Lonati PA Pregnolato F Spaggiari L Besutti G. Complement activation predicts negative outcomes in COVID-19: The experience from Northen Italian patients. Autoimmun Rev. (2022) 103232. doi: 10.1016/j.autrev.2022.103232, PMID: 36414219 PMC9675082

[17] Moore A Ansari M McKeigue P Skerka C Hayward C Rudan I. Genetic influences on plasma CFH and CFHR1 concentrations and their role in susceptibility to age-related macular degeneration. Human Molecular Genetics (2013) 22(23):4857–4869. doi: 10.1093/hmg/ddt336, PMID: 23873044 PMC3820139

[18] Ferluga J Kouser L Murugaiah V Sim RB Kishore U. Potential influences of complement factor H in autoimmune inflammatory and thrombotic disorders. Mol Immunol. (2017) 84:84–106. doi: 10.1016/j.molimm.2017.01.015, PMID: 28216098

[19] Leatherdale A Stukas S Lei V West HE Campbell CJ Hoiland RL. Persistently elevated complement alternative pathway biomarkers in COVID-19 correlate with hypoxemia and predict in-hospital mortality. Med Microbiol Immunol. (2022) 211:37–48. doi: 10.1007/s00430-021-00725-2, PMID: 35034207 PMC8761108

[20] Kouser L Abdul-Aziz M Nayak A Stover CM Sim RB Kishore U. Properdin and factor h: opposing players on the alternative complement pathway “see-saw. Front Immunol. (2013) 4:93. doi: 10.3389/fimmu.2013.00093, PMID: 23630525 PMC3632793

[21] Sim R Day A Moffatt B Fontaine M. 1] Complement factor I and cofactors in control of complement system convertase enzymes. Methods enzymology. (1993) 223:13–35. doi: 10.1016/0076-6879(93)23035-L, PMID: 8271948

[22] Ripoche J Al Salihi A Rousseaux J Fontaine M. Isolation of two molecular populations of human complement factor H by hydrophobic affinity chromatography. Biochem J. (1984) 221:89–96. doi: 10.1042/bj2210089, PMID: 6235808 PMC1144006

[23] Kouser L Abdul-Aziz M Tsolaki AG Singhal D Schwaeble WJ Urban BC. A recombinant two-module form of human properdin is an inhibitor of the complement alternative pathway. Mol Immunol. (2016) 73:76–87. doi: 10.1016/j.molimm.2016.03.005, PMID: 27060503

[24] Al-Mozaini MA Tsolaki AG Abdul-Aziz M Abozaid SM Al-Ahdal MN Pathan AA. Human properdin modulates macrophage: Mycobacterium bovis BCG interaction via thrombospondin repeats 4 and 5. Front Immunol. (2018) 9:533. doi: 10.3389/fimmu.2018.00533, PMID: 29867915 PMC5951972

[25] DiScipio RG. The fractionation of human plasma proteins: I. Affinity Purification Hum Complement Properdin Protein Expression Purification. (1994) 5:164–9. doi: 10.1006/prep.1994.1026, PMID: 8054850

[26] Di Genova C Sampson A Scott S Cantoni D Mayora-Neto M Bentley E. Production, titration, neutralisation, storage and lyophilisation of severe acute respiratory syndrome coronavirus 2 (SARS-CoV-2) lentiviral pseudotypes. Bio-protocol. (2021) 11:e4236–e. doi: 10.21769/BioProtoc.4236, PMID: 34859134 PMC8595416

[27] Cascella M Rajnik M Aleem A Dulebohn SC Di Napoli R. Features, evaluation, and treatment of coronavirus (COVID-19). Treasure Island, FL: Statpearls (2022). 32150360

[28] Afzali B Noris M Lambrecht BN Kemper C. The state of complement in COVID-19. Nat Rev Immunol. (2022) 22:77–84. doi: 10.1038/s41577-021-00665-1, PMID: 34912108 PMC8672651

[29] Chauhan AJ Wiffen LJ Brown TP. COVID-19: a collision of complement, coagulation and inflammatory pathways. J Thromb Haemostasis. (2020) 18:2110–7. doi: 10.1111/jth.14981, PMID: 32608159 PMC7361520

[30] Siggins MK Davies K Fellows R Thwaites RS Baillie JK Semple MG. Alternative pathway dysregulation in tissues drives sustained complement activation and predicts outcome across the disease course in COVID-19. Immunology. (2022) 168:473–92. doi: 10.1111/imm.13585, PMID: 36175370 PMC9537932

[31] Alosaimi B Mubarak A Hamed ME Almutairi AZ Alrashed AA AlJuryyan A. Complement anaphylatoxins and inflammatory cytokines as prognostic markers for COVID-19 severity and in-hospital mortality. Front Immunol. (2021) 2298. doi: 10.3389/fimmu.2021.668725, PMID: 34276659 PMC8281279

[32] Yang R Huang B Ruhan A Li W Wang W Deng Y. Development and effectiveness of pseudotyped SARS-CoV-2 system as determined by neutralizing efficiency and entry inhibition test *in vitro* . Biosafety Health. (2020) 2:226–31. doi: 10.1016/j.bsheal.2020.08.004, PMID: 32864605 PMC7442068

[33] Fu X Tao L Zhang X. Comprehensive and systemic optimization for improving the yield of SARS-CoV-2 spike pseudotyped virus. Mol Therapy-Methods Clin Dev. (2021) 20:350–6. doi: 10.1016/j.omtm.2020.12.007, PMID: 33521163 PMC7823204

[34] Blaum BS Hannan JP Herbert AP Kavanagh D Uhrín D Stehle T. Structural basis for sialic acid–mediated self-recognition by complement factor H. Nat Chem Biol. (2015) 11:77–82. doi: 10.1038/nchembio.1696, PMID: 25402769

[35] Chen JY Cortes C Ferreira VP. Properdin: A multifaceted molecule involved in inflammation and diseases. Mol Immunol. (2018) 102:58–72. doi: 10.1016/j.molimm.2018.05.018, PMID: 29954621 PMC7375857

[36] Mangogna A Varghese PM Agostinis C Alrokayan SH Khan HA Stover CM. Prognostic value of complement properdin in cancer. Front Immunol. (2021) 11:614980. doi: 10.3389/fimmu.2020.614980, PMID: 33542722 PMC7851055

[37] Segers FM Verdam FJ de Jonge C Boonen B Driessen A Shiri-Sverdlov R. Complement alternative pathway activation in human nonalcoholic steatohepatitis. PloS One. (2014) 9:e110053. doi: 10.1371/journal.pone.0110053, PMID: 25299043 PMC4192551

[38] McKenna E Wubben R Isaza-Correa JM Melo AM Mhaonaigh AU Conlon N. Neutrophils in COVID-19: Not innocent bystanders. Front Immunol. (2022) 2548. doi: 10.3389/fimmu.2022.864387, PMID: 35720378 PMC9199383

[39] Del Valle DM Kim-Schulze S Huang H-H Beckmann ND Nirenberg S Wang B. An inflammatory cytokine signature predicts COVID-19 severity and survival. Nat Med. (2020) 26:1636–43. doi: 10.1038/s41591-020-1051-9, PMID: 32839624 PMC7869028

[40] Liu QQ Cheng A Wang Y Li H Hu L Zhao X. Cytokines and their relationship with the severity and prognosis of coronavirus disease 2019 (COVID-19): a retrospective cohort study. BMJ Open. (2020) 10:e041471. doi: 10.1136/bmjopen-2020-041471, PMID: 33257492 PMC7705426

[41] Huang J Hume AJ Abo KM Werder RB Villacorta-Martin C Alysandratos K-D. SARS-CoV-2 infection of pluripotent stem cell-derived human lung alveolar type 2 cells elicits a rapid epithelial-intrinsic inflammatory response. Cell Stem Cell. (2020) 27:962–73. e7. doi: 10.1016/j.stem.2020.09.013, PMID: 32979316 PMC7500949

[42] Kircheis R Haasbach E Lueftenegger D Heyken WT Ocker M Planz O. NF-κB pathway as a potential target for treatment of critical stage COVID-19 patients. Front Immunol. (2020) 11:598444. doi: 10.3389/fimmu.2020.598444, PMID: 33362782 PMC7759159

[43] Davies DA Adlimoghaddam A Albensi BC. The effect of COVID-19 on NF-κB and neurological manifestations of disease. Mol Neurobiology. (2021) 58:4178–87. doi: 10.1007/s12035-021-02438-2, PMID: 34075562 PMC8169418

[44] Milani D Caruso L Zauli E Al Owaifeer AM Secchiero P Zauli G. p53/NF-kB balance in SARS-coV-2 infection: from OMICs, genomics and pharmacogenomics insights to tailored therapeutic perspectives (COVIDomics). Front Pharmacol. (2022) 13. doi: 10.3389/fphar.2022.871583, PMID: 35721196 PMC9201997

[45] Rahman MM McFadden G. Modulation of NF-κB signalling by microbial pathogens. Nat Rev Microbiol. (2011) 9:291–306. doi: 10.1038/nrmicro2539, PMID: 21383764 PMC3611960

[46] Yang L Xie X Tu Z Fu J Xu D Zhou Y. The signal pathways and treatment of cytokine storm in COVID-19. Signal transduction targeted Ther. (2021) 6:1–20. doi: 10.1038/s41392-021-00679-0, PMID: 34234112 PMC8261820

[47] Nazerian Y Vakili K Ebrahimi A Niknejad H. Developing cytokine storm-sensitive therapeutic strategy in COVID-19 using 8P9R chimeric peptide and soluble ACE2. Front Cell Dev Biol. (2021) 9. doi: 10.3389/fcell.2021.717587, PMID: 34540833 PMC8446510

[48] Carcaterra M Caruso C. Alveolar epithelial cell type II as main target of SARS-CoV-2 virus and COVID-19 development via NF-Kb pathway deregulation: A physio-pathological theory. Med Hypotheses. (2021) 146:110412. doi: 10.1016/j.mehy.2020.110412, PMID: 33308936 PMC7681037

[49] Pandey A Mishra AK. From innate immunity to inflammation: A primer on multiple facets of NF-κB signaling in COVID-19. Physiologia. (2022) 2:34–45. doi: 10.3390/physiologia2020004

[50] Kandasamy M. NF-κB signalling as a pharmacological target in COVID-19: potential roles for IKKβ inhibitors. Naunyn-Schmiedeberg’s Arch Pharmacol. (2021) 394:561–7. doi: 10.1007/s00210-020-02035-5, PMID: 33394134 PMC7780215

[51] Dinarello CA. Overview of the IL-1 family in innate inflammation and acquired immunity. Immunol Rev. (2018) 281:8–27. doi: 10.1111/imr.12621, PMID: 29247995 PMC5756628

[52] DeDiego ML Nieto-Torres JL Regla-Nava JA Jimenez-Guardeño JM Fernandez-Delgado R Fett C. Inhibition of NF-κB-mediated inflammation in severe acute respiratory syndrome coronavirus-infected mice increases survival. J virology. (2014) 88:913–24. doi: 10.1128/JVI.02576-13, PMID: 24198408 PMC3911641

[53] Siu K-L Yuen K-S Castaño-Rodriguez C Ye Z-W Yeung M-L Fung S-Y. Severe acute respiratory syndrome coronavirus ORF3a protein activates the NLRP3 inflammasome by promoting TRAF3-dependent ubiquitination of ASC. FASEB J. (2019) 33:8865. doi: 10.1096/fj.201802418R, PMID: 31034780 PMC6662968

[54] Conti P Ronconi G Caraffa A Gallenga C Ross R Frydas I. Induction of pro-inflammatory cytokines (IL-1 and IL-6) and lung inflammation by Coronavirus-19 (COVI-19 or SARS-CoV-2): anti-inflammatory strategies. J Biol Regul Homeost Agents. (2020) 34:327–31. 10.23812/CONTI-E 32171193

[55] Jamilloux Y Henry T Belot A Viel S Fauter M El Jammal T. Should we stimulate or suppress immune responses in COVID-19? Cytokine and anti-cytokine interventions. Autoimmun Rev. (2020) 19:102567. doi: 10.1016/j.autrev.2020.102567, PMID: 32376392 PMC7196557

[56] Martinon F Burns K Tschopp J. The inflammasome: a molecular platform triggering activation of inflammatory caspases and processing of proIL-β. Mol Cell. (2002) 10:417–26. doi: 10.1016/S1097-2765(02)00599-3, PMID: 12191486

[57] Ferreira AC Soares VC de Azevedo-Quintanilha IG Dias S Fintelman-Rodrigues N Sacramento CQ. SARS-CoV-2 engages inflammasome and pyroptosis in human primary monocytes. Cell Death discovery. (2021) 7:1–12. doi: 10.1038/s41420-021-00428-w, PMID: 33649297 PMC7919254

[58] Cauchois R Koubi M Delarbre D Manet C Carvelli J Blasco VB. Early IL-1 receptor blockade in severe inflammatory respiratory failure complicating COVID-19. Proc Natl Acad Sci. (2020) 117:18951–3. doi: 10.1073/pnas.2009017117, PMID: 32699149 PMC7430998

[59] Chen G Wu D Guo W Cao Y Huang D Wang H. Clinical and immunological features of severe and moderate coronavirus disease 2019. J Clin Invest. (2020) 130:2620–9. doi: 10.1172/JCI137244, PMID: 32217835 PMC7190990

[60] Qin C Zhou L Hu Z Zhang S Yang S Tao Y. Dysregulation of immune response in patients with coronavirus 2019 (COVID-19) in Wuhan, China. Clin Infect diseases. (2020) 71:762–8. doi: 10.1093/cid/ciaa248, PMID: 32161940 PMC7108125

[61] Vaidya SA Korner C Sirignano MN Amero M Bazner S Rychert J. Tumor necrosis factor α is associated with viral control and early disease progression in patients with HIV type 1 infection. J Infect diseases. (2014) 210:1042–6. doi: 10.1093/infdis/jiu206, PMID: 24688071 PMC4215080

[62] Seo SH Webster RG. Tumor necrosis factor alpha exerts powerful anti-influenza virus effects in lung epithelial cells. J virology. (2002) 76:1071–6. doi: 10.1128/JVI.76.3.1071-1076.2002, PMID: 11773383 PMC135862

[63] Karki R Sharma BR Tuladhar S Williams EP Zalduondo L Samir P. Synergism of TNF-α and IFN-γ triggers inflammatory cell death, tissue damage, and mortality in SARS-CoV-2 infection and cytokine shock syndromes. Cell. (2021) 184:149–68. e17. doi: 10.1016/j.cell.2020.11.025, PMID: 33278357 PMC7674074

[64] Luo P Liu Y Qiu L Liu X Liu D Li J. Tocilizumab treatment in COVID-19: a single center experience. J Med virology. (2020) 92:814–8. doi: 10.1002/jmv.25801, PMID: 32253759 PMC7262125

[65] Lee JS Shin E-C. The type I interferon response in COVID-19: implications for treatment. Nat Rev Immunol. (2020) 20:585–6. doi: 10.1038/s41577-020-00429-3, PMID: 32788708 PMC8824445

[66] Wang N Zhan Y Zhu L Hou Z Liu F Song P. Retrospective multicenter cohort study shows early interferon therapy is associated with favorable clinical responses in COVID-19 patients. Cell Host Microbe. (2020) 28:455–64. e2. doi: 10.1016/j.chom.2020.07.005, PMID: 32707096 PMC7368656

[67] Cesta MC Zippoli M Marsiglia C Gavioli EM Mantelli F Allegretti M. The role of interleukin-8 in lung inflammation and injury: Implications for the management of COVID-19 and hyperinflammatory acute respiratory distress syndrome. Front Pharmacol. (2022) 12:3931. doi: 10.3389/fphar.2021.808797, PMID: 35095519 PMC8790527

[68] Folkesson H Matthay M Hebert C Broaddus V. Acid aspiration-induced lung injury in rabbits is mediated by interleukin-8-dependent mechanisms. J Clin Invest. (1995) 96:107–16. doi: 10.1172/JCI118009, PMID: 7615779 PMC185178

[69] Zeng Z-Y Feng S-D Chen G-P Wu J-N. Predictive value of the neutrophil to lymphocyte ratio for disease deterioration and serious adverse outcomes in patients with COVID-19: a prospective cohort study. BMC Infect Diseases. (2021) 21:1–6. doi: 10.1186/s12879-021-05796-3, PMID: 33461497 PMC7812552

[70] Thevarajan I Nguyen TH Koutsakos M Druce J Caly L van de Sandt CE. Breadth of concomitant immune responses prior to patient recovery: a case report of non-severe COVID-19. Nat Med. (2020) 26:453–5. doi: 10.1038/s41591-020-0819-2, PMID: 32284614 PMC7095036

[71] Alturaiki WH. Evaluation of CC chemokine ligand 5 (CCL5) chemokine, interleukin 5 (IL-5) cytokine, and eosinophil counts as potential biomarkers in Saudi patients with chronic asthma during sandstorms. Cureus. (2020) 12. doi: 10.7759/cureus.7809, PMID: 32467785 PMC7249775

[72] Marques RE Guabiraba R Russo RC Teixeira MM. Targeting CCL5 in inflammation. Expert Opin Ther targets. (2013) 17:1439–60. doi: 10.1517/14728222.2013.837886, PMID: 24090198 PMC7103722

[73] Patterson BK Seethamraju H Dhody K Corley MJ Kazempour K Lalezari J. CCR5 inhibition in critical COVID-19 patients decreases inflammatory cytokines, increases CD8 T-cells, and decreases SARS-CoV2 RNA in plasma by day 14. Int J Infect Diseases. (2021) 103:25–32. doi: 10.1016/j.ijid.2020.10.101, PMID: 33186704 PMC7654230

[74] Varghese PM Tsolaki AG Yasmin H Shastri A Ferluga J Vatish M. Host-pathogen interaction in COVID-19: Pathogenesis, potential therapeutics and vaccination strategies. Immunobiology. (2020) 225:152008. doi: 10.1016/j.imbio.2020.152008, PMID: 33130519 PMC7434692

