# Supplementary material for: Differential modulation of SARS-CoV-2 infection by complement factor H and properdin
Source: Front Immunol. 2025 Aug 15;16:1620229. doi: 10.3389/fimmu.2025.1620229 (PMC12395051; doi:10.3389/fimmu.2025.1620229)
Supplement: Supplementary Figure 1 — Characterisation of purified complement proteins. The immunoreactivity of the purified complement proteins was assessed using western blotting. A PVDF membrane was employed and probed at RT for 1h with specific antibodies (1:1,000 dilution). The antibodies used were rabbit-anti-human Properdin polyclonal antibodies, and mouse-anti-human FH monoclonal antibody (MRCOX23). Additionally, a protein ladder spanning a range of 250 to 10 kDa was included. Following the primary antibody incubation, the membrane was subsequently incubated with secondary antibodies, either goat anti-rabbit IgG HRP-conjugate or goat anti-mouse IgG HRP-conjugate (1:1,000 dilution), for 1h at RT. The resulting bands corresponding to the respective proteins were observed. For FH was observed at ~155 kDa (A), Properdin exhibited a band ~55 kDa (B) and TSR4 + 5 was at ~55 kDa (C), after developing the colour using 3,3′-diaminobenzidine (DAB) substrate. [file DataSheet1.docx]

**Supplementary File**

**Differential Modulation of SARS-CoV-2 Infection by Complement Factor H and Properdin**

Uday Kishore^1,2*^, Praveen M Varghese^3^, Chandan Kumar^4^, Susan Idicula-Thomas^4^, Martin Mayora Neto^5^, Anthony G. Tsolaki^6^, Pretty Ponanachan^1^, Khaled Masmoudi^7^, Basel Al-Ramadi^8,9^, Manu Vatish^10^, Taruna Madan^11^, Nigel Temperton^5^, Nazar Beirag^3,6^

^1^Department of Veterinary Medicine (CAVM), United Arab Emirates University, Al Ain, U.A.E.

^2^Zayed Centre for Health Sciences, United Arab Emirates University, Al Ain, U.A.E.

^3^Department of Clinical Microbiology, Umea University, Umea, Sweden

^4^Biomedical Informatics Centre, ICMR-National Institute for Research in Reproductive and Child Health, Mumbai 400012, Maharashtra, India

^5^Viral Pseudotype Unit, Medway School of Pharmacy, University of Kent and Greenwich, Kent ME4 4TB, UK

^6^Biosciences, College of Health, Medicine and Life Sciences, Brunel University London, Uxbridge UB8 3PH, United Kingdom

^7^Department of Integrative Agriculture (CAVM), United Arab Emirates University, Al Ain, U.A.E.

^8^Department of Medical Microbiology and Immunology, College of Medicine and Health Sciences, United Arab Emirates University, Al Ain, U.A.E.

^9^ASPIRE Precision Medicine Research Institute Abu Dhabi, United Arab Emirates University, Al Ain, U.A.E.

^10^Nuffield Department of Women's and Reproductive Health, University of Oxford, U.K.

^11^Department of Innate Immunity, ICMR-National Institute for Research in Reproductive and Child Health, Mumbai, India

^11^Department of Biosciences, College of Health, Medicine and Life Sciences, Brunel University of London, Uxbridge UB8 3PH, United Kingdom

**^*^**Corresponding authors: Uday Kishore ([ukishore@hotmail.com](mailto:ukishore@hotmail.com); uday.kishore@uaeu.ac.ae)

**Running Title:** Factor H and properdin differentially modulate SARS-CoV-2 Infection

**Keywords**: Innate Immune System; Complement System; Alternative Pathway; Properdin; Factor H; SARS-CoV-2; CoVID-19; Cytokine response.

# Supplementary Figures


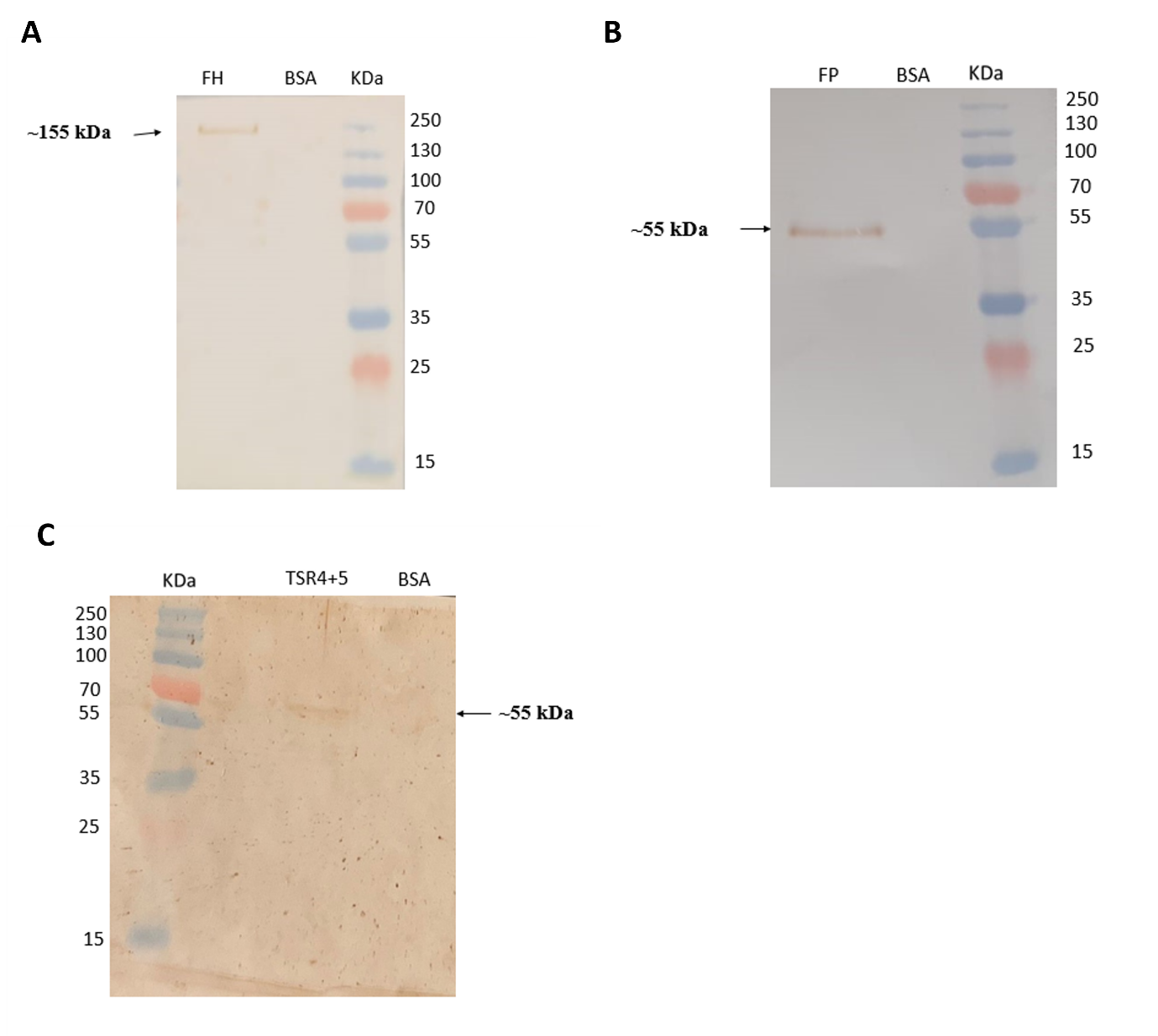


**Figure S1: Characterization of purified complement proteins.**

The immunoreactivity of the purified complement proteins was assessed using western blotting. A PVDF membrane was employed and probed at room temperature for 1 h with specific antibodies (1:1,000 dilution). The antibodies used were rabbit-anti-human Properdin polyclonal antibodies, and mouse-anti-human FH monoclonal antibody (MRCOX23). Additionally, a protein ladder spanning a range of 250 to 10 kDa was included. Following the primary antibody incubation, the membrane was subsequently incubated with secondary antibodies, either goat anti-rabbit IgG HRP-conjugate or goat anti-mouse IgG HRP-conjugate (1:1,000 dilution), for 1 h at room temperature. The resulting bands corresponding to the respective proteins were observed. For Factor H was observed at ~ 155 kDa (A), Properdin exhibited a band ~ 55 kDa (B) and TSR4+5 was at ~ 55 kDa (C), after developing the colour using 3,3′-diaminobenzidine (DAB) substrate.


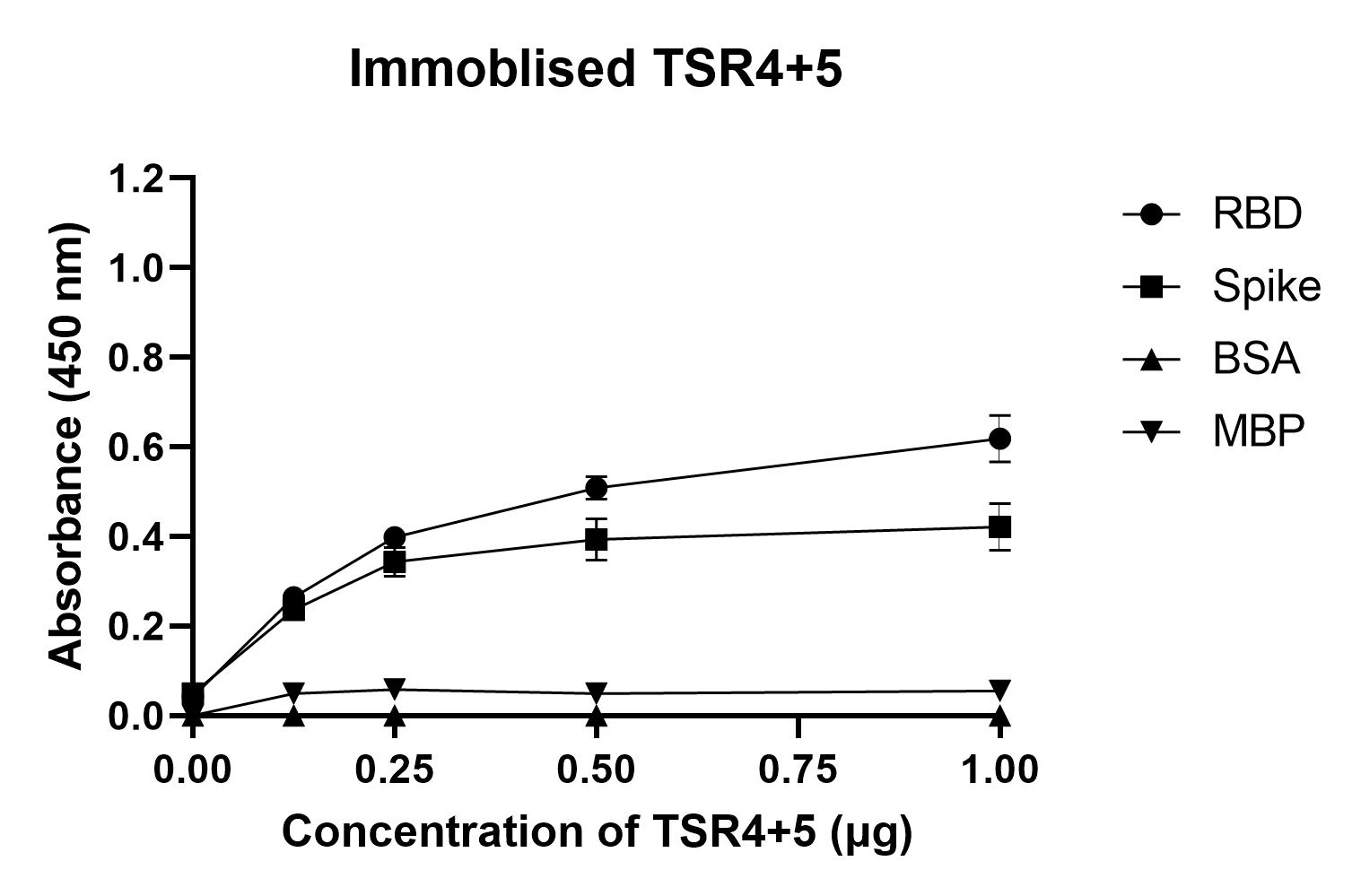


**Figure S2: SARS-CoV-2 interacted with TSR4+5 via its S Protein RBD.**

TSR4+5 bound both SARS-CoV-2 Spike and RBD proteins in a dose-dependent manner. Decreasing concentration of immobilised TSR4+5 (1, 0.5, 1.25, and 0 μg/well) were coated in a 96-well plate using Carbonate-Bicarbonate (CBC) buffer, pH 9.6 at 4°C overnight. After washing out the excess CBC buffer with PBS, three times, a constant concentration of virus proteins (1 μg/well) was added to corresponding wells, followed by incubation at 37°C for 2h. After washing out the unbound proteins, the wells were probed with rabbit anti-SARS-CoV-2 Spike (1:5000; 100 μ/well). MBP and BSA were used as negative control.

**
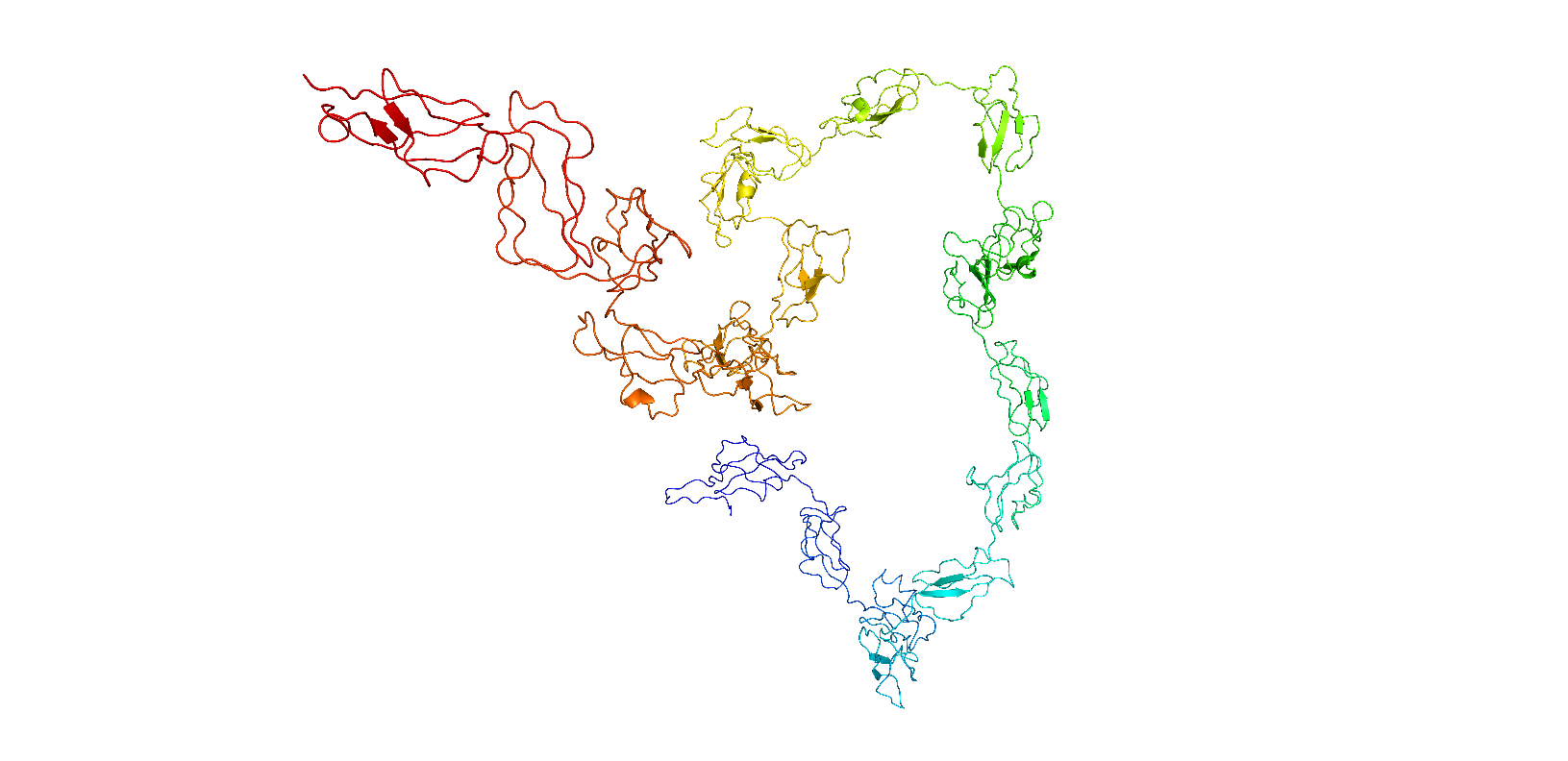
**

**Figure S3:** The cartoon representation of the full-length 3D structure of FH consists of 20 short consensus/complement repeat (SCR) domains. This structure was sub-optimal due to several unfolded regions and large, extended structures that were unsuitable for docking. The structure was modeled using ModellerCartoon representation of modelled FH structure. Full-length 3D structure of FH was modelled using Modeller10.1.


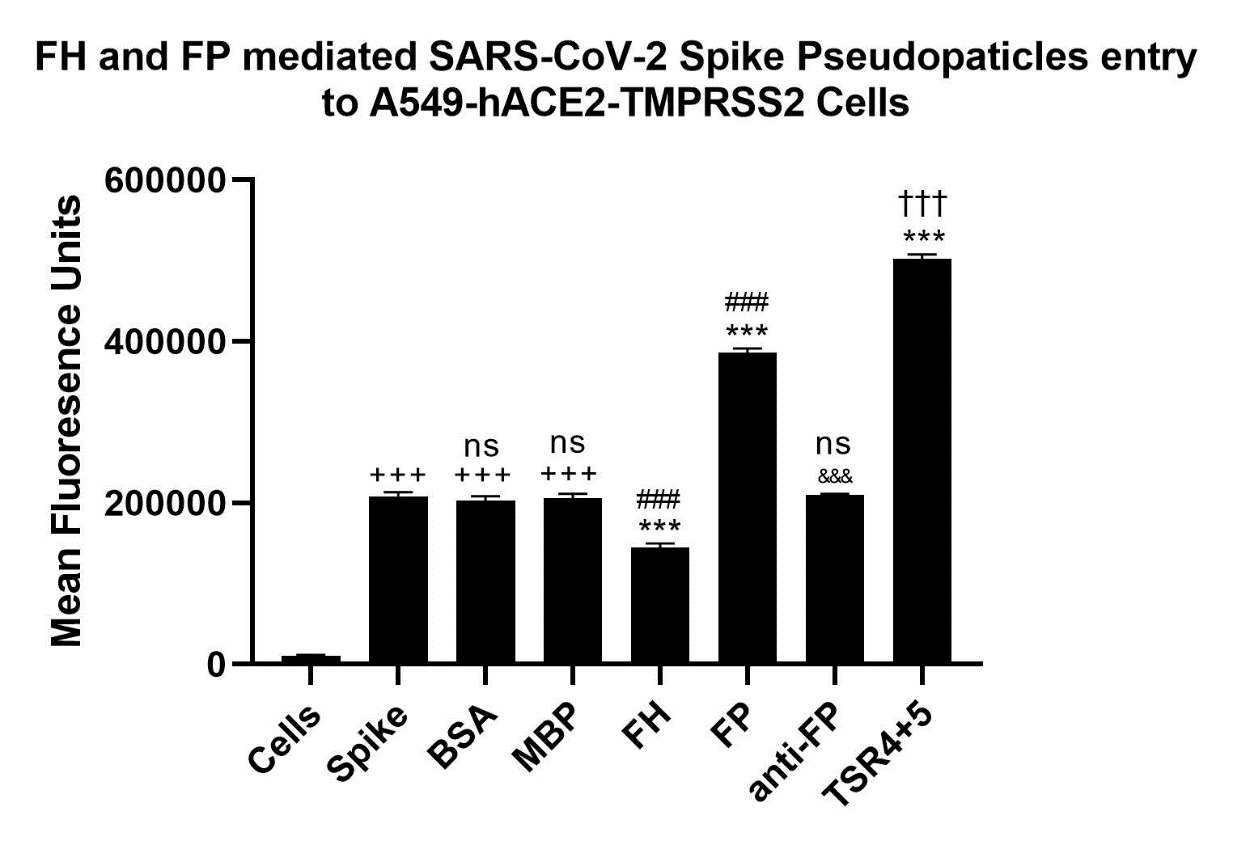


**Figure S4: FH, FP and TSR 4+5 modulate SARS-CoV-2 pseudoparticle entry into A549-hACE2+TMPRSS2 cells.**

Luciferase reporter activity of FH, FP or TSR 4+5 (20 μg/mL) pre-treated cells transduced with SARS-CoV-2 lentiviral pseudoparticles for 24h was measured. The assay was conducted in triplicates, and error bars represent ± SEM. The statistical significance of Spike, BSA or MBP treated cells were compared to untreated cells was determined by using the two-way ANOVA (^+++^p < 0.05). Similarly, the statistical significance of FH or FP pre-treated cells challenged with SARS-CoV-2 lentiviral pseudoparticles was also determined by using the two-way ANOVA by comparing to cells that were only challenged with the pseudotypes (***p < 0.05), or to BSA pretreated cells challenged with the pseudotypes (^###^p < 0.05). Since the recombinant TSR4+5 is tagged with MBP, the statistical significance of TSR4+5 pre-treated cells challenged with the pseudotypes was assessed using the two-way ANOVA by comparing to cells that were pretreated with MBP and then challenged with the pseudotypes (^†††^p < 0.05). Notably, anti-FP has statistically significant (^&&&^p < 0.05) reduced the viral entry effect of FP treated cells, while no significantly found as compared to cells treated with Spike or BSA (ns > 0.05). The results clearly show that FH pretreatment significantly reduces viral entry, while pretreatment with either FP or TSR 4+5 significantly increases viral entry. Finally, no statistically significant difference using the two-way ANOVA was identified between BSA or MBP pre-treated cells when as compared to cells directly challenged with the pseudotypes (ns > 0.05).


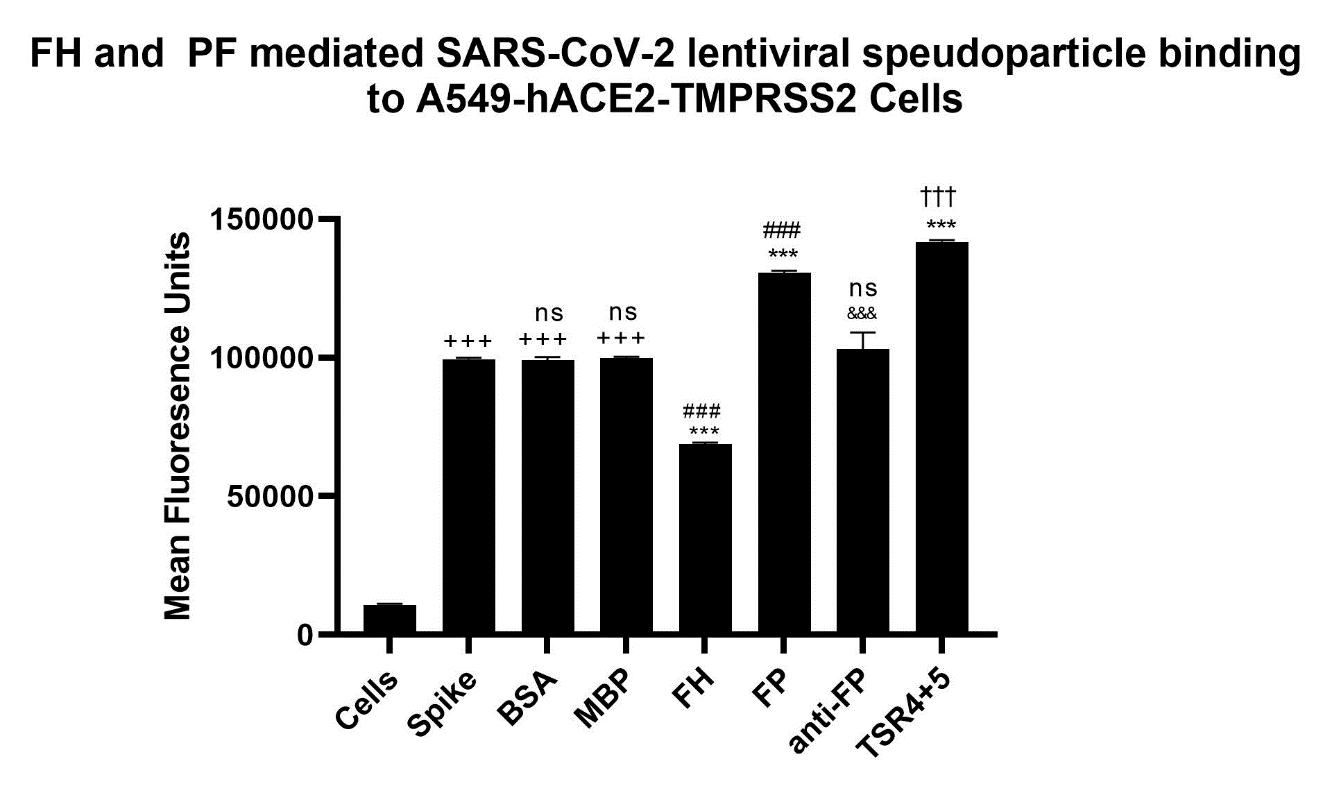


**Figure S5: Binding of FH or PF pre- treated SARS-CoV-2 pseudoparticles to A549-hACE2+TMPRSS2 cells.**

SARS-CoV-2 lentiviral pseudoparticles were used to transduce A549-hACE2+TMPRSS2 cells, which were pre-incubated with FH or FP (20 µg/mL). The wells were probed with rabbit anti-SARS-CoV-2 spike (1:200) polyclonal antibodies after being washed and fixed with 1% v/v paraformaldehyde for 1 min. Viral entry assay was conducted in triplicates, and error bars represent ± SEM. Statistical significance was determined using the two-way ANOVA (^+++^p < 0.05) for cells treated with Spike, BSA, or MBP as compared to untreated cells. For cells pre-treated with FH or FP and then challenged with SARS-CoV-2 lentiviral pseudoparticles, significance was compared to cells only challenged with pseudotypes (***p < 0.05) or to BSA pre-treated cells subsequently challenged with pseudotypes (^###^p < 0.05). Recombinant TSR4+5 (tagged with MBP), the significance of TSR4+5 pre-treated cells challenged with pseudotypes was evaluated against cells pre-treated with MBP followed by a pseudotype challenge (^†††^p < 0.05). Importantly, anti-FP has statistically significant (^&&&^p < 0.05) reversed the binding effect of FP treated cells, whereas no significantly found when compared to cells treated with Spike or BSA (ns > 0.05). These findings revealed that FH pretreatment reduced viral binding, whereas FP or TSR 4+5 pretreatment enhanced it. No significant difference was observed between BSA or MBP pre-treated cells and those directly challenged with pseudotypes (ns > 0.05).
